# Integrative Model of the Immune Response to a Pulmonary Macrophage Infection: What Determines the Infection Duration?

**DOI:** 10.1371/journal.pone.0107818

**Published:** 2014-09-18

**Authors:** Natacha Go, Caroline Bidot, Catherine Belloc, Suzanne Touzeau

**Affiliations:** 1 UR341 MIA, INRA, Jouy-en-Josas, France; 2 LUNAM Université, Oniris, INRA UMR 1300 BioEpAR, Nantes, France; 3 UMR1355 ISA, INRA, Université Nice Sophia Antipolis, CNRS, Sophia Antipolis, France; 4 BIOCORE, Inria, Sophia Antipolis, France; University of Catania, Italy

## Abstract

The immune mechanisms which determine the infection duration induced by pathogens targeting pulmonary macrophages are poorly known. To explore the impact of such pathogens, it is indispensable to integrate the various immune mechanisms and to take into account the variability in pathogen virulence and host susceptibility. In this context, mathematical models complement experimentation and are powerful tools to represent and explore the complex mechanisms involved in the infection and immune dynamics. We developed an original mathematical model in which we detailed the interactions between the macrophages and the pathogen, the orientation of the adaptive response and the cytokine regulations. We applied our model to the Porcine Respiratory and Reproductive Syndrome virus (PRRSv), a major concern for the swine industry. We extracted value ranges for the model parameters from modelling and experimental studies on respiratory pathogens. We identified the most influential parameters through a sensitivity analysis. We defined a parameter set, the reference scenario, resulting in a realistic and representative immune response to PRRSv infection. We then defined scenarios corresponding to graduated levels of strain virulence and host susceptibility around the reference scenario. We observed that high levels of antiviral cytokines and a dominant cellular response were associated with either short, the usual assumption, or long infection durations, depending on the immune mechanisms involved. To identify these mechanisms, we need to combine the levels of antiviral cytokines, including 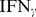, and 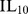. The latter is a good indicator of the infected macrophage level, both combined provide the adaptive response orientation. Available PRRSv vaccines lack efficiency. By integrating the main interactions between the complex immune mechanisms, this modelling framework could be used to help designing more efficient vaccination strategies.

## Introduction

Respiratory pathogens, which enter the body through the mucosal surfaces of the respiratory tract, are responsible for local inflammation and tissue damages [1], [2]. They initiate the infection and the immune response. The first interaction between the pathogen and the immune system involves the innate immune system. This first line of defence, which includes epithelial surfaces, inflammation process, complement system and innate cells, provides an immediate but non-specific response. The innate cells mainly consist of the pulmonary macrophages, the dendritic cells and the natural killers. Macrophages and dendritic cells phagocyte the pathogens, whereas the natural killers destroy the host infected cells. If pathogens successfully evade the innate response, a second layer of protection is provided by the adaptive immune system, which is activated by the innate response and confers specific long-lasting protective immunity to the host. The adaptive immune system mainly involves the cellular, the humoral and the regulatory responses. The cellular effectors destroy the infected cells, whereas the humoral effectors release antibodies, which are responsible for the neutralisation of free viral particles. The regulatory response mainly inhibits the adaptive response. Innate and adaptive immune cells synthesise cytokines, small proteins which regulate the immune mechanisms in complex ways.

The best strategy to control the severity of respiratory pathogens is to limit the inflammation while maintaining an efficient immune response. Some pathogens, such as influenza viruses, *Mycobacterium tuberculosis* or the Porcine Reproductive and Respiratory Syndrome virus, replicate in the cells of the respiratory tract, including pulmonary macrophages. They hinder the immune functions of the macrophages and consequently reduce the efficacy of the immune response. With these pathogens, activated macrophages (i) either phagocyte and destroy the pathogen, or are infected and excrete the pathogen; (ii) produce cytokines that promote the migration of immune cells to the infection site; (iii) synthesise cytokines that regulate the adaptive immunity; (iv) express antigen proteins on their cell surface that activate the adaptive response. In turn, the adaptive cell effectors and cytokines regulate the immune functions of macrophages. However, the influence of macrophage–pathogen interactions on the immune response has been poorly studied and needs more insight [1]–[4]. The two major reasons are that the innate mechanisms are very difficult to explore by experimentation *in vivo* and that they have been considered as having little impact compared to the adaptive response.

Here, we were interested in identifying the immune mechanisms which determine the infection duration induced by pathogens targeting pulmonary macrophages. The immune response is a highly complex system involving numerous interactions between cells and cytokines. An additional level of complexity is due to the between-host and between-pathogen variability. Pathogens use multiple strategies, that vary among pathogens but also among strains, resulting in various virulence levels. The host response depends on the host genotype or housing conditions, resulting in various susceptibility levels to a given pathogen. Consequently, to explore the impact of pathogens targeting pulmonary macrophages, it is indispensable to integrate the various immune mechanisms and to take into account the variability in pathogen virulence and host susceptibility.

In this context, mathematical models are powerful tools to represent and explore the complex mechanisms involved in the infection and immune dynamics [3], [5]. They complement experimentation. On the one hand, they are based on experimental data. On the other hand, they can be used to test biological hypotheses or assess the impact of control strategies, which would not be feasible or would be too expensive by experimentation. They can also guide experimentation by identifying key parameters or mechanisms that need further exploration. Mathematical models have been developed to explore the immune and infection dynamics for various human and animal diseases. However, very few models represent the innate mechanisms explicitly and macrophage–pathogen interactions need to be better represented in models [6]. Several models describe pathogens targeting macrophages, such as influenza viruses [5]–[7], *Mycobacterium tuberculosis* [8], [9], or Porcine Respiratory and Reproductive Syndrome virus [10]. These models focused more on the adaptive than on the innate response, which was fairly simplified or even missing. In particular, none of these models included the macrophage and natural killer immune functions explicitly and innate the cytokine regulations were simplified. Moreover, none took into account the regulatory adaptive response.

So we proposed an original model of the immune response to a virus infecting pulmonary macrophages in the lung. We considered with particular attention the macrophage–virus interactions. We highly detailed the mechanisms of the innate response and the cytokine regulations. We included the cellular, the humoral and the regulatory orientation of the adaptive response, as well as their main functions. We represented the interactions between innate and adaptive components. We applied our model to the Porcine Respiratory and Reproductive Syndrome virus (PRRSv). PRRSv is a major concern for the swine industry, as it is responsible for significant economic losses worldwide [11], [12]. This pathogen is of particular interest because: (i) it exhibits a strong tropism for the pulmonary macrophages [11]–[14]; (ii) it induces a prolonged viremia thanks to its ability to hamper the immune response [11], [12], [15]; and (iii) the infection and immune dynamics are highly variable between hosts and viral strains. Depending on the studies, various components of the immune response have been highlighted as having an impact on PRRSv infection duration: (i) the macrophage permissiveness and excretion rate; (ii) the levels of antiviral and immuno-modulatory cytokines; and (iii) the balance between the cellular, humoral and regulatory responses [16]. We used our integrative model to identify the immune mechanisms determining the infection duration and to explore the relevance of these three assumptions, taking into account the variability in pathogen virulence and host susceptibility.

First, we built our model by synthesising knowledge on the immune mechanisms from published studies on PRRSv. Experimental studies on PRRSv are numerous, but cannot provide all our model parameter values. So we compiled data from the literature by reviewing experimental and modelling studies on pathogens targeting pulmonary macrophages and obtained large value ranges for our model parameters. We explored the influence of these parameters on the viral and macrophage dynamics by a sensitivity analysis. We then identified a parameter set resulting in realistic infection and immune dynamics. Finally, we explored the influence of host susceptibility and viral virulence on the infection outcome and we identified the associated immune mechanisms.

## Methods

In this section, we first present the dynamic model and its calibration, based on literature data. We then describe the sensitivity analysis method used to quantify the influence of model parameters on outputs of interest, among which the viral titer. Finally, we define scenarios which represent the variability in host susceptibility and strain virulence, in order to assess the impact of this variability on the model outputs.

### Model description

**Figure 1:**
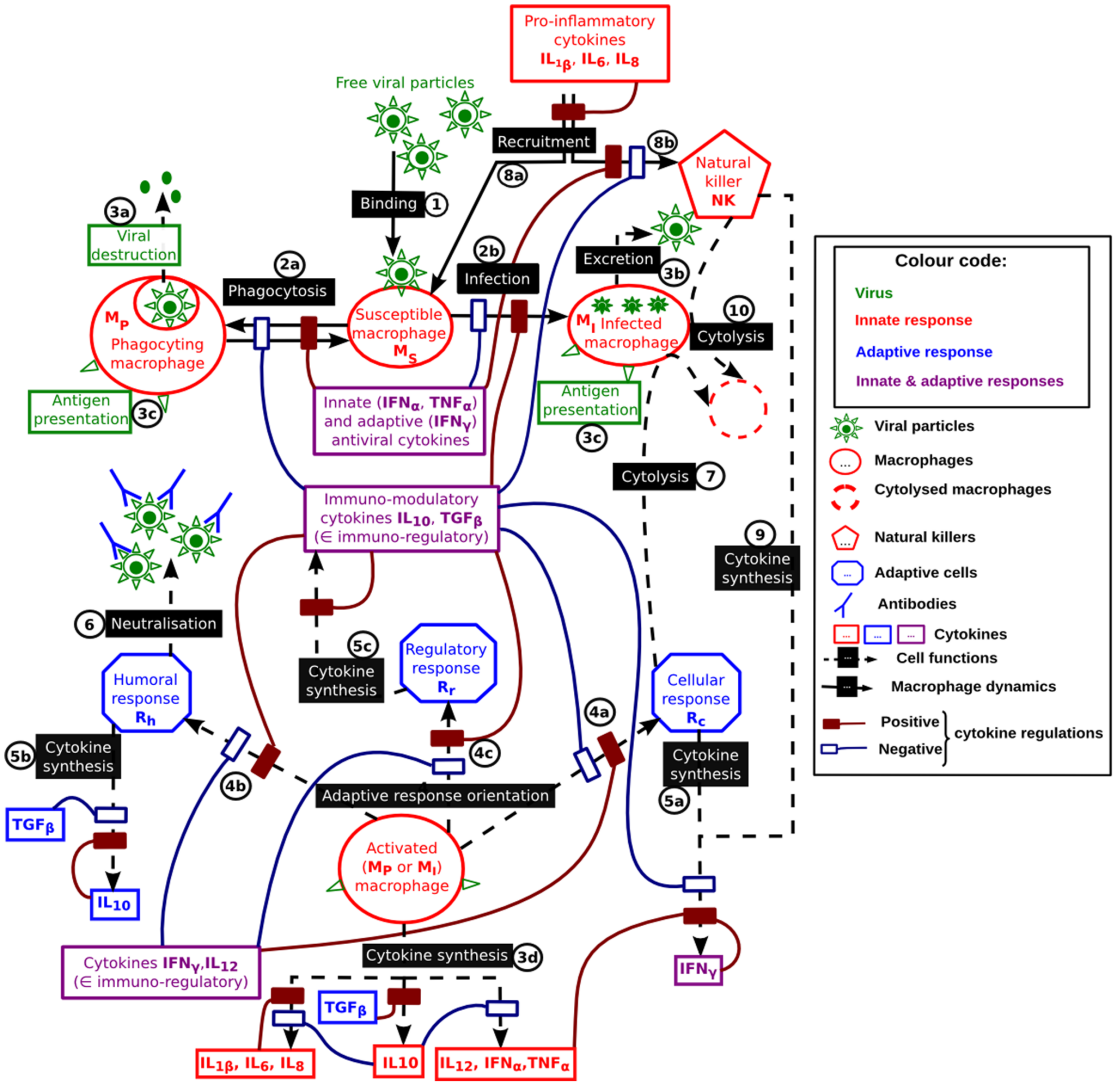
Functional diagram of the immune response to a virus targeting macrophages. Interactions between macrophages and virus (1) result in macrophage activation by either phagocytosis (2a, amplified by antiviral cytokines and inhibited by immuno-modulatory cytokines) or macrophage infection (2b, amplified by immuno-modulatory cytokines and inhibited by antiviral cytokines) releasing viral particles (3b). The activated macrophages initiate the adaptive response (4a–c). 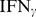 and 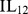 orient the adaptive response towards the cellular response (4a), whereas immuno-modulatory cytokines orient the response towards the humoral and regulatory responses (4b–c). The cellular response and the natural killers are responsible for the destruction of infected cells by cytolysis (7 & 10, respectively). The humoral response is responsible for the viral neutralisation through antibodies (6). The recruitment of susceptible macrophages and natural killers is amplified by the pro-inflammatory cytokines (8a & 8b, respectively). Cytokines are produced by activated macrophages (3d), natural killers (9) and adaptive cells (4a–c). These syntheses are regulated by various cytokines.

We built a deterministic dynamic model of ordinary differential equations to simulate the infection and immune dynamics induced by a pathogen targeting pulmonary macrophages in the lung. The functional diagram of the model appears in Figure 1. We selected the immune components and their interactions from current knowledge on the immune mechanisms induced by pathogens targeting pulmonary macrophages. Our modelling assumptions are detailed and justified in the complete model description given in Appendix S1. In particular, the cytokine regulations and syntheses represented in our model, as well as the related literature references, are summarised in Table S1 and Table S2 respectively.

The model is characterised by 18 state variables: the free viral particles (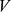); five effectors of the innate response: four macrophage states and the natural killers (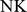); three effectors of the adaptive response and nine cytokines. A macrophage can either be susceptible (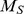), phagocyting (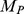), or infected; in this latter case, it is either latent (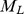) or excreting the virus (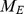). For the adaptive response, the effectors represent the regulatory (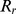), humoral (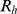) and cellular (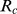) responses. The nine cytokines included are the major pro-inflammatory (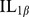, 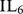, 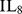), the innate antiviral (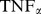, 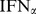) and the immuno-regulatory (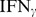, 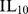, 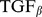, 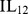) cytokines. 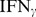 also exhibits an antiviral function. 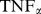 is generally considered as a pro-inflammatory cytokine, but we were here more interested is its antiviral function. The model describes the evolution over time of the state variable concentrations in the lung.

**Figure 2:**
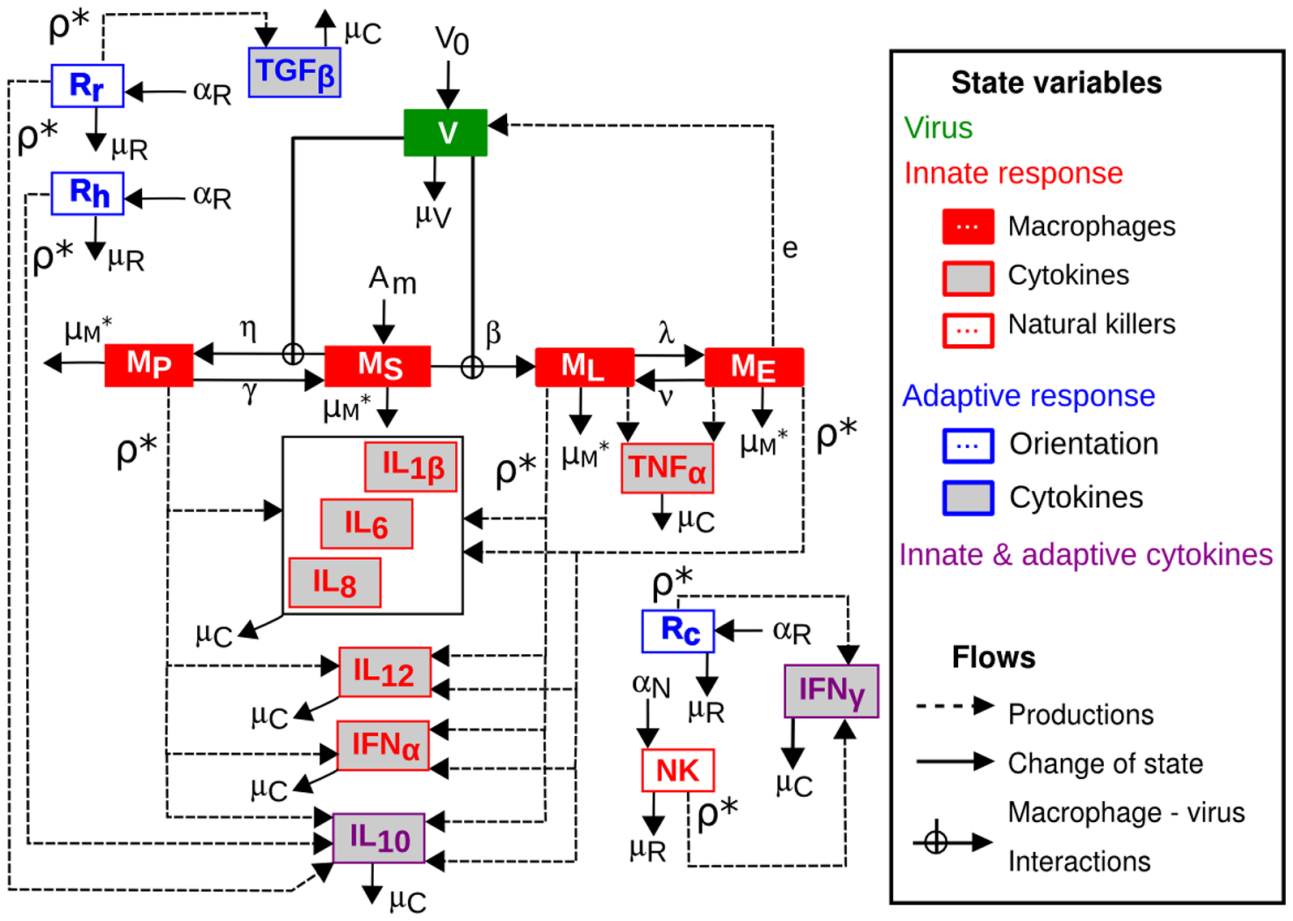
Conceptual model: state variables and flows without regulations. The state variables consist of: the free viral particles (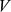); the susceptible (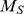), phagocyting (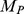), latent (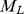) and excreting (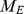) macrophages; the natural killers (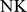); the cellular (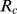), humoral (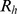) and regulatory (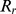) adaptive cells; the pro-inflammatory cytokines (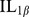, 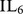 & 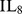; grouped in the box), the innate antiviral cytokines (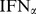 & 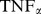) and the immuno-regulatory cytokines (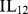, 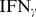, 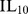 & 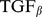). The flows represented are: the inoculation of free viral particles (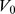); the recruitment of susceptible macrophages (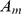); the activation of natural killers (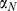) and cells of the adaptive response (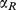); the decay of the free viral particles (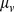), the macrophages (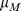
*), the natural killers (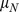), the adaptive cells (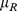) and the cytokines (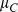); the macrophage state changes, *i.e.* phagocytosis (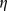 and 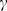), infection (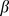) and transient excretion (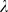 and 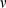); the excretion of free viral particles by infected macrophages (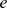) and the cytokine syntheses by activated immune cells (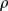
*). For the sake of readability, the cytokine and cell regulations and not drawn and some parameter notations (marked with *) are simplified.

The main processes that drive the evolution of these state variables and that are integrated in the model are: the phagocytosis of the viral particles by the macrophages (rate 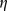); the macrophage infection by the virus (rate 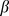); the excretion of free viral particles by the infected macrophages (rate 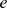); the recruitment (rate 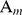) and decay/migration of the macrophages (rates 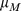); the activation (rates 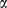) and decay/migration of the other effectors (rates 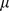); the cytokine productions by the immune cells (rates 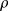) and their decay (rates 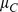); the cytokine regulations (functions 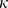). Figure 2 gives a schematic representation of the model (without regulations). Parameter descriptions and values are synthesised in Table 1. A complete description of the model and the corresponding equations is given in Appendix S1. Here we describe the main components of the model, illustrated by a few representative equations.

**Table 1:** Model parameters.

Parameter	Description	Tested values			Reference value	Unit	References
*Macrophage dynamics*							
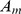	recruitment rate of 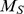	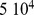	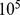	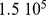	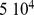	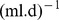	[21], [62]
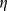	phagocytosis rate	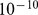	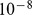	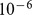	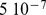	ml/d*	[68]
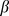	infection rate	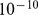	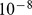	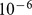	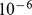	ml/d*	[14]
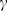	1/phagocytosis duration	24	48	96	96	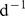	[23]
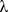	1/duration of 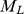 state	6	12	24	6	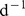	[14]
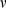	1/duration of 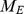 state	6	12	24	6	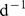	–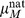	natural death rate	0.1	0.2	0.3	0.2	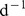	[22]
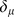	over-mortality rate of 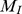	0.9	1	1.1	1.1	no unit	–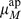	apoptosis rate by 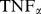	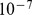	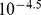	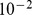	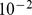	ml/(pg.d)	[69]
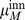	cytolysis rate of 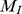 by NK	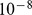	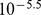	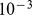	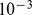	ml/d	[69]
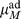	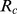 cytolysis rate of 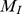 by	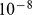	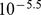	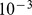	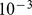	ml/d	[69]
*Virus dynamics*							
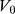	initial viral inoculation	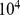	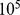	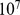	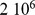	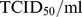	[38], [39]
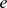	excretion rate	0.1	1	10	0.2	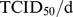	–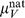	natural death rate	0.1	0.2	0.3	0.2	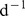	–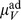	neutralisation rate by 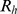	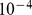	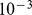	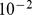	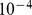	ml/d	–
*Adaptive cell (* 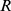 *) and natural killer (* 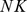 *) dynamics*							
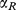	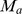 activation rate of 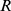 by	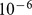	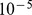	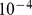	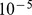	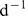	[21]
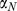	activation rate of 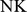	0.1	1	10	10	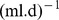	[21]
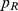	proliferation rate of 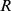	0.05			0.05	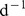	–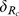	death rate of 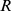 by AICD	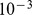	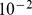	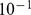	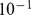	ml/d	–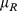	natural death rate	0.01	0.03	0.05	0.05	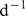	–
*Cytokine dynamics*							
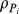	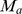 synthesis rate of 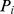 by	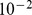	10	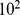	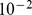	pg/d	–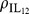	synthesis rate of 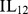 by 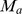	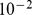	10	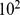	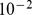	pg/d	–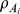	synthesis rate of 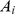 by 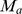	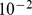	10	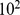	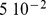	pg/d	–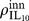	synthesis rate of 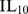 by 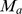	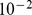	10	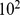	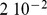	pg/d	–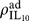	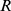 synthesis rate of 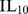 by	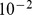	10	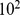	2	pg/d	–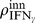	synthesis rate of 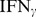 by NK	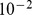	10	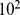	10	pg/d	–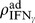	synthesis rate of 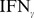 by 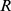	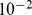	10	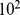	10	pg/d	–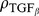	synthesis rate of 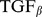 by 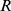	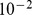	10	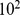	10	pg/d	–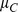	natural death rate	10	20	40	20	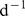	[22]
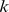	half-saturation concentration	5	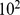	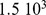	30	pg/ml	[22]
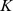	saturation factor	0.5	1	1.5	1.5	no unit	–

The minimal and maximal values tested were issued from the literature when we found some information, otherwise they were assumed (–). Macrophages: susceptible (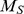), latent (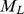), excreting (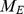), infected (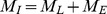), phagocyting (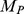), activated (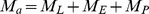). Adaptive cells 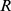: cellular (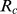), humoral (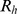) and regulatory (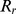) effectors.

* The unit of 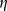 and 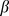 is given for the macrophage equation and is different in the virus equation (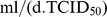); nevertheless, the parameter values are the same since we considered that the phagocytosis and macrophage infection consume one 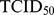 of virus per macrophage.

**Figure 3:**
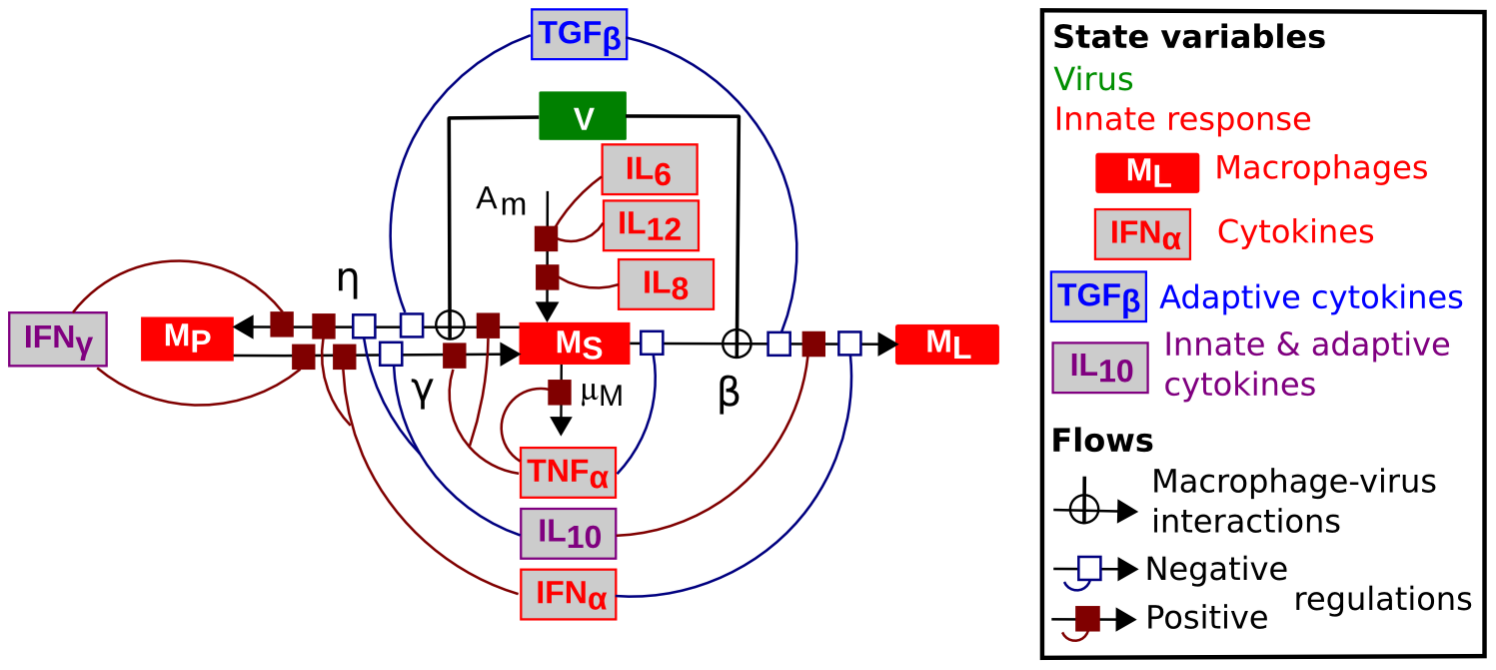
Susceptible macrophage dynamics with cytokine regulations. The state variables represented are: the free viral particles (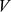); the susceptible (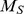), phagocyting (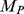) and latent (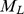) macrophages; the pro-inflammatory cytokines (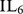 & 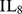), the innate antiviral cytokines (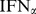 & 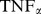) and the immuno-regulatory cytokines (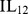, 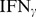, 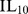 & 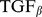). All processes that impact the susceptible macrophages are included: recruitment (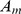), decay (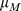, simplified notation), phagocytosis (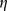 and 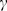) and infection (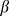); their positive and negative regulations by cytokines are also drawn.

When a free viral particle encounters a susceptible macrophage (1 in Figure 1), it can either be phagocyted (rate 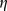, 2a in Figure 1), resulting in viral destruction (3a in Figure 1), or it can infect the cell (rate 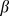, 2b in Figure 1), resulting in viral replication (3b in Figure 1). The phagocytosis is amplified by antiviral cytokines (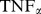, 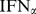 and 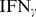) and inhibited by immuno-modulatory cytokines (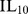 and 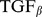, 2a in Figure 1). The infection (linked to the macrophage permissiveness) is amplified by 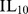 and inhibited by innate antiviral cytokines (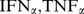) and 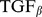 (2b in 1). Phagocyting macrophages revert to a susceptible status after viral destruction (rate 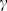); it is amplified by the antiviral cytokines and inhibited by 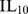 (2a in Figure 1). Activated macrophages (infected or phagocyting macrophages) produce pro-inflammatory cytokines (3d in Figure 1), which amplify the recruitment of susceptible macrophages (inflow 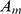, 8a in Figure 1) [4], [17]–[19]. Finally, susceptible macrophages undergo natural decay (rate 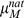) and 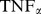-induced apoptosis (rate 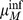) [20]. The resulting susceptible macrophage dynamics is shown in Equation (1) and Figure 3.
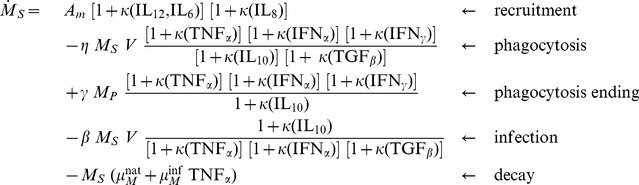
(1)


The cytokine environment is not static in our model, as we explicitely represented the evolution of the cytokine concentrations over time. Cytokines are produced by activated immune cells. In turn, they modulate the cellular functions through their recognition by specific receptors, inducing cascaded reactions within the cells. The higher the cytokine concentration, the stronger the effect. However, there is a limited number of cytokine receptors on the cell surface, so the effect saturates above a given cytokine concentration. We formalised the cytokine effects by a Michaelis–Menten function (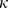) of the cytokine concentration (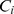) [8], [21], [22] as follows:
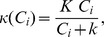



where 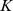 represents the saturation factor and 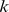 the half-saturation concentration. A cytokine can have three possible effects listed below on a given flow (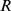).

- Activation: 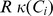. The flow is only possible in the presence of the cytokine and it increases with the cytokine concentration.
- Amplification: 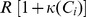. The flow increases with the cytokine concentration.
- Inhibition: 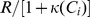. The flow decreases with the cytokine concentration.

Regulations often involve several cytokines (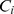 and 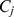), which can act

- either independently: 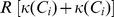 for an activation, 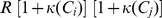 for an amplification, or 
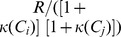
 for an inhibition;
- or in synergy: 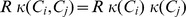 for an activation, 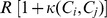 for an amplification, or 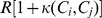 for an inhibition.

For example, the recruitment of susceptible macrophages (8a in Figure 1) is amplified by three cytokines, as shown in Equation (1): two act in synergy (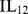 and 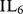) and the third one acts independently (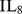).

The dynamics of natural killers, given by Equation (2), offers a more complex example of cytokines acting independently and in synergy. We represented the dynamics of activated natural killers and only included the regulations by the most influential cytokines [4], [19], [23], [24]. The recruitment of natural killers from the bloodstream (rate 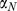, 8b in Figure 1) is activated by pro-inflammatory cytokines: 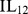 and 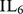 co-activate the recruitment, whereas 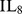 acts independently. Natural killers are then activated by 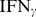 and 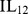, whereas 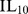 inhibits the activation. They are submitted to natural death or/and migration (rate 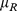). Activated natural killers destroy infected cells (10 in Figure 1) and synthesise 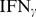 (9 in Figure 1) [4], [19], [23], [24].
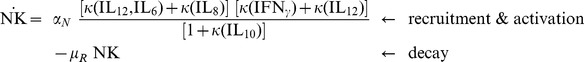
(2)


Activated macrophages present the viral antigens to the adaptive cells (3c in Figure 1). The subsequent orientation of the adaptive response depends on the immuno-regulatory cytokines (4a – c in Figure 1). We represented the adaptive response by three effectors corresponding to the three main orientations: cellular, humoral and regulatory responses [1], [4], [13], [25]–[31]. As for the natural killers, we only represented the dynamics of the activated effectors. Based on the model proposed by Yates *et al.* for the regulation of T helper cell populations [31], we synthesised the dynamics of each adaptive effector by three steps: activation by activated macrophages (rate 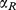), proliferation (rate 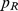) and decay. We represented the regulations of the activation and proliferation steps by the most influential cytokines: 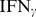, 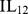, 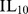 and 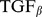 (assumptions and references detailed in Appendix S1). The decay includes the natural decay (rate 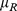) and the Activation Induced Cell Death (AICD) induced by the interaction with a type 1 T helper cell from the 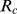 compartment (basic rate 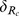) [31].

- Cellular response: 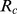 represents the type 1 T helper cells and the cytotoxic lymphocytes. Its dynamics is described in Equation (3). Activation is amplified by 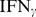 and 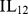 and inhibited by 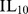; proliferation is activated by 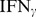 and 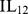 and inhibited by 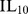 and 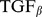 (4a in Figure 1). 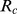 synthesises 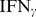 (5a in Figure 1) and destroys infected macrophages (7 in Figure 1).

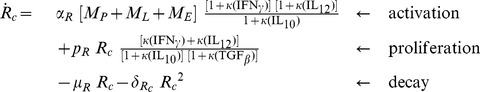
(3)
- Humoral response: 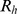 represents the type 2 T helper cells, the B lymphocytes and the antibodies. Activation is amplified by 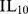 and inhibited by 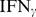 and 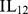; proliferation is activated by 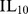 and inhibited by 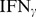, 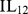 and 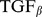 (4b in Figure 1). 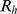 synthesises 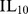 (5b in Figure 1) and neutralises free viral particles through antibodies (6 in Figure 1).
- Regulatory response: 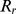 represents the regulatory T cells. Activation is amplified by 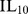 and 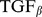 and inhibited by 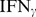 and 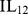; proliferation is activated by 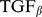 and inhibited by 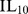, 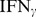 and 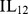 (4c in Figure 1). 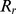 synthesises 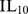 and 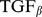 (5c in Figure 1).

#### Simulations

The model was implemented in Scilab 5.3.3 (http://www.scilab.org/). For all simulations, the initial conditions were set to represent an initial viral inoculation in a PRRSv-naive host and were chosen as follows: 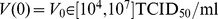 for the viral titer; 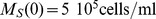 for the susceptible macrophages; all remaining variables were set to zero. The model parameters are summarised in Table 1.

### Model calibration

Published experimental data on PRRSv infection (reviewed in [11], [12], [16], [32]–[37]) are highly heterogeneous and differ on: (i) the monitoring duration, (ii) the measured immune components, (iii) the viral strain, (iv) the pig genotype. Moreover, among the variables included in our model, only a few were monitored in each experimental study and there were few measures over time. Consequently, based on these data, classical parameter estimation methods were not suitable to calibrate our model and we had to design an *ad hoc* procedure.

The first step of the calibration procedure was to synthesise data from experimental infections to identify the variation ranges of our model parameters. When PRRSv studies could not provide parameter values, we reviewed models applied to tuberculosis and influenza. The value ranges obtained for the model parameters and the corresponding references are given in Table 1 (ranges defined by the minimum and maximum tested values). The second step was to explore the parameter space defined by these value ranges. We used a design of experiments which is described in the Sensitivity analysis section below. The simulations resulting from this exploration exhibited very contrasted outputs (Figures S2–S4). So the third step was to define the characteristics of the infection and immune dynamics corresponding to a realistic response to PRRSv infection. We chose to represent an average response as our reference scenario (S0). This step is detailed below. Finally, the fourth step was to select a parameter set corresponding to this reference scenario. We used the sensitivity analyses presented below to focus first on the most sensitive parameters, *i.e.* parameters which had the greatest impact on the model outputs.

For the reference scenario, we chose to represent the infection of a weaned pig by a single PRRSv inoculation. Weaned pigs are supposed to be naive to PRRSv and to have lost their maternal immunity. In experimental PRRSv infection studies, the inoculation dose ranged between 4 and 7 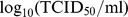 [38], [39]; we chose an inoculation dose of 6.3 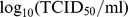. PRRSv infection usually lasts between 28 to 42 days in the blood [12], [16], [40] and around 56 days in the lung [12]. However, the infection duration is highly variable between pigs and viral strains and can be higher than 200 days [16], [41]. So we chose an infection duration in the lung of around 70 days. Few quantitative data are available for the immune dynamics. The cytokine levels are highly variable between studies [11], [13] and poorly documented in the lung. Their magnitude ranges between 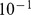 and 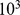 pg/ml. 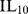 levels in response to PRRSv infection and other respiratory pathogens are similar. They are higher than the levels of pro-inflammatory, antiviral (innate and adaptive) and other immuno-regulatory (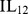, 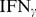 and 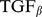) cytokines. Without infection, macrophage concentrations in the lung were observed around 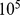 cells/ml. To our knowledge, only one experimental study tracked infected macrophages, which peaked during the first days of PRRSv infection at around 40% among all macrophages [39]. Little is known about the phagocyting macrophages, except that the phagocyting state is transient and that PRRSv promotes macrophage infection over phagocytosis [42], [43]. Reported levels of natural killers during PRRSv infection were low compared to other respiratory pathogens [15], [33]. The humoral response to PRRSv infection is similar to other respiratory pathogens, whereas the cell-mediated immunity is delayed and weak. The regulatory response has been poorly studied and results are controversial [13], [44]–[46]. Moreover, the orientation of the adaptive response varied considerably between studies. Consequently, we chose a balanced adaptive response orientation for our reference scenario.

### Sensitivity analysis

We were interested in identifying the most influential parameters on the infection dynamics thanks to a global sensitivity analysis. Consequently, the first two outputs selected were the viral titer (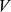) and the percentage of infected macrophages among the total concentration of macrophages (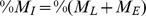). We were also interested in characterising the phagocytosis activity, which directly limits the macrophage infection. The phagocytosis is a transient macrophage state, which explains why, whatever the parameter combination selected in the parameter ranges, the percentage of phagocyting macrophages (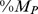) was low compared to the percentage of infected macrophages (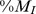) at any time during the course of infection (Figure **S1**). However, it does not mean that the phagocytosis activity was necessarily low. We compared the phagocytosis flow (susceptible macrophages becoming phagocyting macrophages per unit of time) and the infection flow (susceptible macrophages becoming infected macrophages per unit of time) during the course of infection. Depending on the parameter values, the phagocytosis inflow was higher or lower than the infection inflow (Figure **S1**). Consequently, the cumulative number of phagocyting macrophages (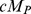), which corresponds to the phagocytosis flow integrated over time, is a good representation of the phagocytosis activity. So we selected this variable as the third output of interest. We used a design of experiments to define which simulations to run. The resulting outputs were analysed to produce the sensitivity indices, which quantify the influence of the parameters on the model outputs. We used the R software, version 3.0.2, (http://www.r-project.org/) for these analyses.

**Figure 4:**
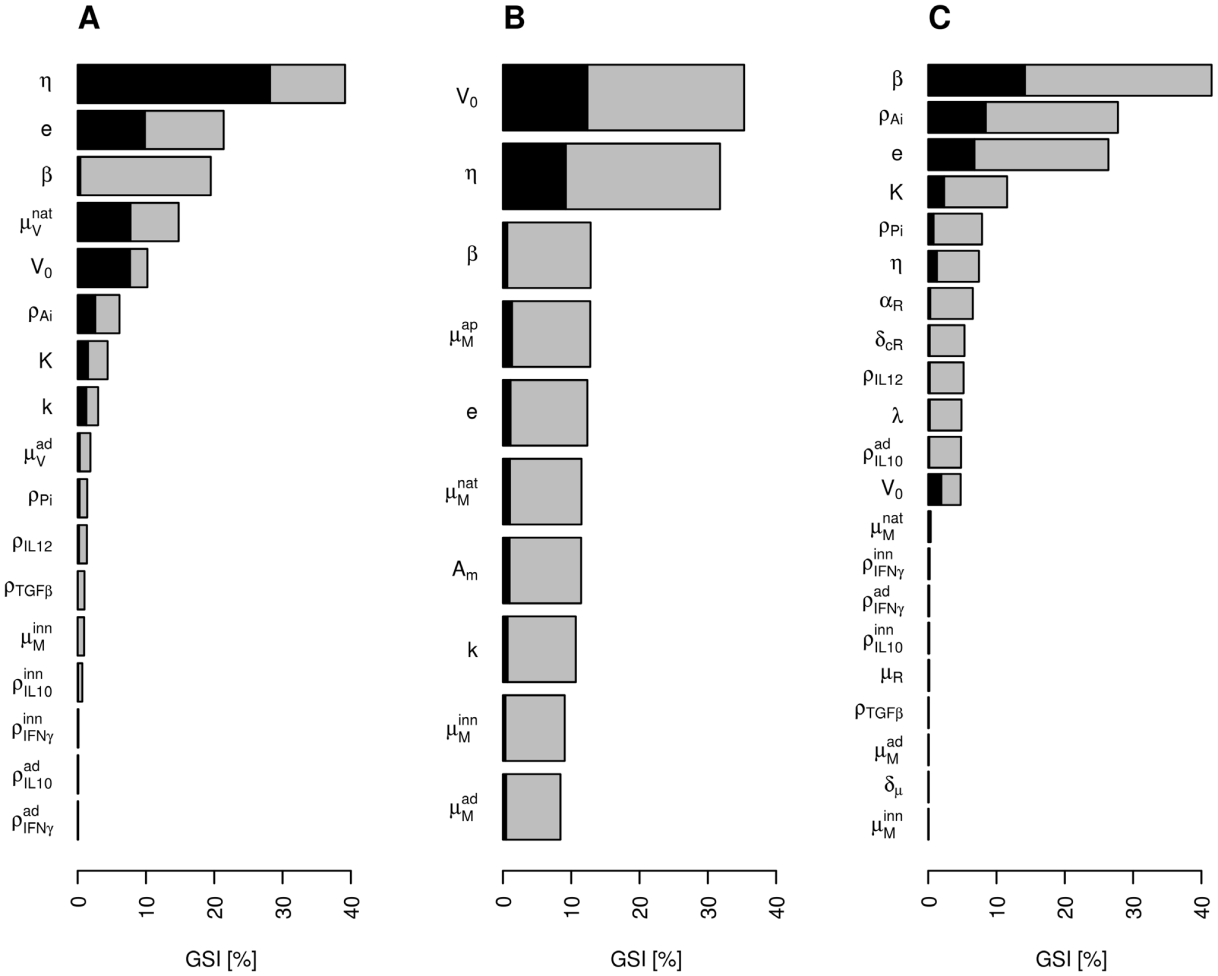
Generalised sensitivity indices (GSI) for the three outputs of interest. **A**: Viral titer 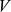 (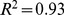). **B**: Cumulative number of phagocyting macrophages 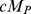 (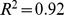). **C**: Percentage of infected macrophages among all macrophages 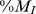 (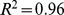). Total GSI (bars) are represented for an output-dependent selection of influential parameters. For each parameter, the total GSI is split into main parameter effect (black bar) and the sum of two-parameter interactions involving the parameter (grey bar). 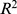 corresponds to the fraction of output variance explained by the parameters. NB: As the two-parameter interactions are counted for both parameters, the sum of the total GSI is higher than 100%.

We selected 30 among the 31 model parameters for the sensitivity analysis. We did not include the proliferation rate of the adaptive effectors (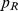), because the combination of high 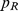 and high 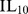 synthesis rates led to the explosion of the 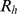 and 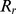 dynamics, which resulted in a numerical integration failure of the model. For each of the 30 parameters, we chose to test three values among the value range identified in the calibration procedure: the lower and upper bounds of the range, as well as an intermediate value (Table 1). Testing all parameter combinations, *i.e.* a complete factorial design, would have required 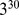 simulations, which was not feasible. Consequently, fractional factorial designs were used instead. A preliminary analysis was conducted to estimate the main effects of the 30 parameters on the model outputs, without taking into account the interactions between parameters. A fractional design of size 243, determined as the minimum size to correctly estimate the main effects, was implemented: 243 parameter combinations were defined and the corresponding simulations were performed and analysed. From this preliminary analysis, the ten most influential parameters on each of the three outputs were identified. We then performed a sensitivity analysis on each output, aiming at estimating the main effects and two-parameter interactions of the corresponding ten most influential parameters, to which we potentially added extra parameters assumed to have an impact on the corresponding output. For instance, we added the macrophage mortality rates for the 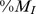 output. We selected 17 parameters for the viral titer, 10 parameters for the cumulative 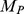 and 21 parameters for the percentage of infected macrophages (Figure 4). The smallest design that correctly estimates the main effects and two-parameter interactions for 21 parameters (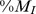 output) requires 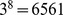 parameter combinations. We chose to use the same design size for all outputs, so 6561 simulations were performed and analysed for each of the three outputs. The Planor R package (http://cran.r-project.org/web/packages/planor/index.html) was used to construct the fractional designs.

Sensitivity indices were calculated for each parameter on each output in the preliminary analysis (30 parameters 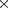 3 outputs) and the subsequent analyses taking into account two-factor interactions. Sensitivity indices quantify the fraction of output variance among simulations explained by the variation of each parameter within its value range [47]. Our model outputs being time-dependent variables, we used a method adapted to multivariate outputs, which is based on a decomposition of the variable using a principal component analysis (PCA) [48]. As a result of the PCA, an inertia proportion is attributed to each component. It represents the variability among simulations carried by the component. Moreover, each simulation is given a "score" on each component, a scalar which represents the projection of the simulation on the component. Then, for each component, an ANOVA is performed on these scores to estimate the influence of each parameter on the output. The sensitivity index associated with each term, main effect of a parameter or interaction between parameters, is defined as the ratio between the sum of squares corresponding to that term and the total sum of squares. Finally, a generalised sensitivity index (GSI) is calculated for each term (main effect or interaction) as the the sum of the sensitivity indices corresponding to that term on each PCA component, weighted by the inertia of the component. The total generalised sensitivity index (tGSI) of a parameter is defined as the sum of the sensitivity indices corresponding to this parameter (main effect mGSI plus sum of interactions involving the parameter iGSI). We used the Multisensi R package (http://cran.r-project.org/web/packages/multisensi/index.html) for this analysis.

GSI results are presented below. For each output, key parameters are defined as the most influential parameters for which the cumulative total GSI is higher than 75%.

### Variability in host susceptibility and strain virulence

PRRSv exhibits an important genotypic diversity associated with various virulence levels [13]. The European genotype is less virulent than the American genotype [35], but the virulence also differs among strains within a genotype [49]. The highly virulent strains are associated with a prolonged viremia, a high viral replication rate and a high humoral response [50]. Moreover, the genetic component of the host susceptibility to PRRSv has been demonstrated [51], [52]. Pig susceptibility can also depend on other factors such as herd management. The more susceptible pigs develop prolonged viremia, with low titers of neutralising antibodies [10], [52], probably linked to a high macrophage permissiveness and/or specific cytokine profiles [51].

Both viral virulence and pig susceptibility seem linked to: (i) the virus capacity to infect the cell and replicate, (ii) the host capacity to synthesise antiviral *vs* immuno-modulatory cytokines in response to PRRSv infection, and (iii) the activation and orientation of the adaptive response. Recent studies hypothesise that these variations of the immune dynamics are due to cascaded reactions initiated by the macrophage–virus interactions [33], [34], [49], [51]. Consequently, we focused on the macrophage infection and cytokine synthesis capacities. Both macrophage permissiveness and viral replication impact the cytokine synthesis, which in turn regulates them. Discriminating the respective influence of the macrophage permissiveness and the cytokine synthesis rate is very difficult experimentally, but it can be achieved by a modelling approach. To explore the influence of both mechanisms, scenarios were defined by varying a selection of parameters chosen according to the sensitivity analysis results and to the hypotheses presented above. We tested 19 graduated values of: (i) the macrophage permissiveness, promoting either the phagocytosis (scenarios S0 to S1: S0
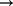
S1), or the macrophage infection and viral excretion (scenarios S0
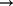
S2); and (ii) the cytokine synthesis rates, promoting either the antiviral cytokine synthesis (scenarios S0
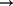
SB), or the immuno-modulatory cytokine synthesis (scenarios S0
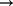
SA). Scenarios are defined in Table 2. Compared to the reference scenario (S0), scenarios S0
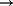
S1 and S0
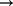
SB correspond to low host susceptibility and strain virulence, whereas scenarios S0
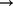
S2 and S0
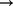
SA correspond to high susceptibility and virulence. The parameter ranges were set to cover the variation range of the viral titer reported in the literature.

**Table 2:** Definition of the host susceptibility and strain virulence scenarios.

	Macrophage permissiveness				Cytokine synthesis capacities				
Scenarios	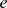	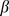	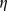	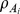	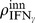	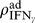	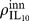	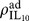	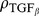
S1	0.15	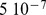	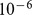	**0.05**	**10**	**10**	**0.02**	**2**	**10**
SB	**0.2**	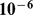	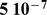	0.5	100	100	0.005	0.5	2.5
**S0**	**0.2**	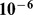	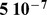	**0.05**	**10**	**10**	**0.02**	**2**	**10**
SA	**0.2**	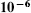	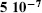	0.005	0.1	0.1	0.08	8	40
S2	0.25	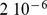	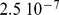	**0.05**	**10**	**10**	**0.02**	**2**	**10**

Scenarios S1 and S2 differ from the reference scenario S0 by their respectively low and high macrophage permissiveness. Scenarios S1
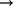
S2 correspond to 19 intermediate scenarios (including S0) obtained by gradually varying the following parameter values: excretion rate (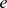), macrophage infection rate (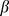) and phagocytosis rate (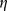). Scenarios SB and SA differ from the reference scenario S0 by their cytokine synthesis capacities: scenario SB promotes antiviral over immuno-modulatory cytokine synthesis, and *vice versa* for scenario SA. Scenarios SB
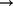
SA correspond to 19 intermediate scenarios (including S0) obtained by varying gradually the synthesis rates of the following cytokines: the innate antiviral cytokines 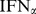 and 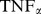 (both 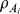); the immuno-regulatory cytokines 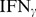 (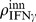 & 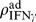), 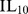 (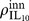 & 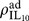) and 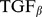 (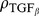). Low/high susceptibility and virulence levels correspond to scenarios with low/high macrophage permissiveness (S1/S2) and scenarios which promote the antiviral/immuno-modulatory cytokine synthesis (SB/SA). The parameter values corresponding to the reference scenario are in boldface.

We used the area under the curve (AUC) to synthesise our model outputs. As the shapes of the immune and viral output curves were similar across the scenarios, characterising each curve by a well-chosen number was appropriate and facilitated the comparisons between scenarios. Choosing the AUC was relevant, as it reflects the entire curve [53]. Relative AUC were defined as percentages of output AUC among a group of outputs.

Several linear regressions were performed to extract trends from our results and facilitate the interpretations. In particular, to highlight the links between immuno-regulatory cytokines and the orientation of the adaptive response, we performed linear regressions between (i) the relative AUC of relevant cytokines and (ii) the relative AUC of the adaptive response effectors (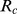, 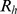 & 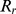). To highlight the immune mechanisms determining the infection duration, we performed linear regressions between (i) the AUC of relevant immune components, which are assumed to have a strong influence on the infection duration in the literature and (ii) the infection duration. We used the R software, version 3.0.2, for these analyses.

The infection duration is defined as the time elapsed between the initial viral inoculation and the virus clearance. In our model, we assumed that there was no more infection when the virus titer was below 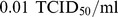.

## Results

### Model calibration and sensitivity analysis

The reference scenario (S0) was characterised by a 72-day infection duration, an infected macrophage peak at 40% of the total macrophage concentration, a balanced adaptive response orientation and high 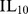 levels compared to antiviral and pro-inflammatory cytokine levels. Its parameter values are given in Table 1 and it is represented in Figue 5 (black curves).

**Figure 5:**
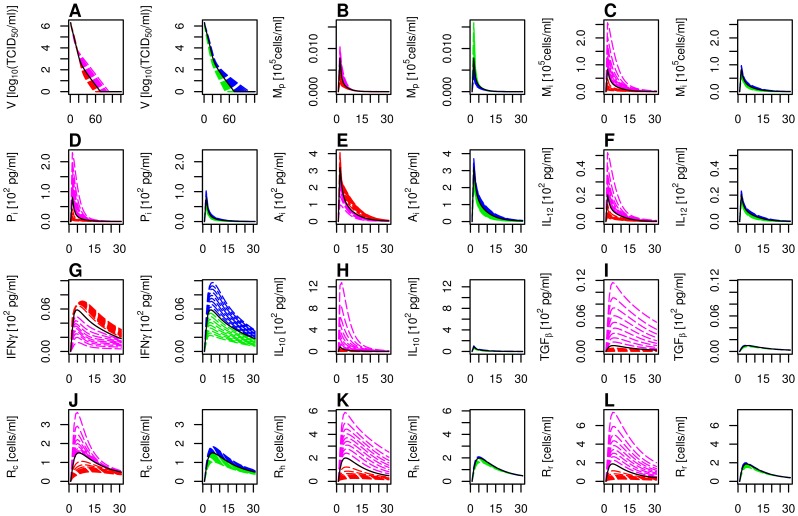
Immune and infection dynamics for variable host susceptibility and strain virulence. Evolution of twelve variables (panels **A** to **L**) during the first 30 days of infection (unless specified). **A**: Viral titer (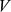, during 120 days). **B**: Phagocyting macrophages (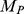). **C**: Infected macrophages (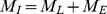). **D**: Pro-inflammatory cytokines (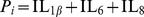). **E**: Innate antiviral cytokines (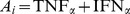). **F**–**I**: Immuno-regulatory cytokines (**F**: 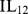, **G**: 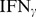, **H**: 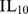 and **I**: 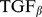). **J**: Adaptive cellular effectors (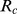). **K**: Adaptive humoral effectors (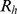). **L**: Adaptive regulatory effectors (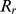). For each variable, the left plot corresponds to scenarios SB
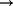
SA, in which the antiviral cytokine synthesis is higher (S0
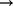
SB, red) or lower (S0
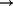
SA, magenta) than in the reference scenario (S0, black). The right plot corresponds to scenarios S1
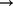
S2, in which the macrophage permissiveness is lower (S0
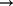
S1, green) or higher (S0
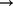
S2, blue) than in the reference scenario (S0, black). Low susceptibility and virulence levels correspond to scenarios which promote the antiviral cytokine synthesis (red) and scenarios with low macrophage permissiveness (green). High susceptibility and virulence levels correspond to scenarios which promote the immuno-modulatory cytokine synthesis (magenta) and scenarios with high macrophage permissiveness (blue) Scenarios are defined in Table 2.

In the preliminary sensitivity analysis, with all 30 parameters but no interactions between parameters, the variance explained by the parameters retained for the main sensitivity analysis on each output was 89% for the viral titer, 89% for the cumulative number of phagocyting macrophages and 70% for the percentage of infected macrophages. The results of the main sensitivity analyses with two-parameters interactions are shown in Figure 4; for each output, the total global sensitivity index defined for each parameter is split into the parameter main effect and its interactions. At least 92% of the total output variance was explained by the parameters and two-parameter interactions for all three outputs. Three key parameters (explaining together more than 75% of the variance) were identified for each output. Their impact is detailed in Table 3. Most of them were involved in macrophage–virus interactions. The infection rate 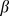 was a key parameter for the three outputs of interest. The excretion rate 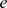 was a key parameter for the viral titer and the percentage of infected macrophages. The phagocytosis rate 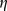 was a key parameter for the viral titer and the cumulative number of phagocyting macrophages. The remaining key parameters were the inoculation dose 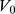 for the cumulative number of phagocyting macrophages and the synthesis rate of innate antiviral cytokines 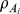 for the percentage of infected macrophages. The main effects of the key parameters ranged between 0.4% (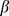 on the viral titer) and 28% (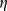 on the viral titer). Key parameters also exhibited high interactions (*e.g.* 27% for interactions involving 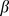 on the percentage of infected macrophages), in particular between two key parameters (results not shown).

**Table 3:** Generalised sensitivity indices and influence of the key parameters on the three outputs of interest.

	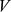			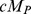			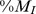		
Key parameters	mGSI	iGSI	Influence	mGSI	iGSI	Influence	mGSI	iGSI	Influence
Initial inoculation 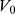	8	3	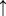	**12**	**23**	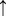	2	3	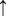
Excretion rate 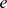	**10**	**12**	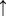	1	11	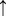	**7**	**20**	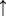
Infection rate 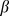	**0.4**	**19**	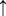	**0.6**	**12**	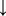	**14**	**27**	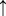
Phagocytosis rate 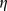	**28**	**11**	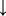	**9**	**23**	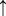	1	6	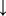
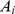 synthesis rate 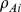	3	4	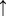	–	–	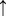	**8**	**19**	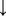

The outputs are the viral titer (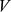), the cumulative number of phagocyting macrophages (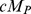), and the percentage of infected macrophages among all macrophages (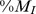). Three key parameters were identified for each output (corresponding GSI in bold). For each parameter and each output, the generalised sensitivity index of the parameter main effect (mGSI, in %) and of the sum of two-parameter interactions involving the parameter (iGSI, in %) are given. Increasing the parameter value can induce an increase (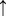) or decrease (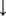) of the output.

The initial inoculation dose 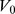 was a key parameter for the cumulative number of phagocyting macrophages (tGSI = 35%), but neither for the viral titer (tGSI = 11%), nor for the percentage of infected macrophages (tGSI = 5%). This result can be explained by the fact that the phagocytosis activity mostly occurs during the first days of the infection, whereas the viral titer and infected macrophages are impacted all along the infection course (Figures S2–S4).

The infection rate 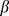 had less impact on the viral titer variability (tGSI = 19.4%, mGSI = 0.4%) than the phagocytosis rate 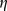 (tGSI = 39%, mGSI = 28%) and the excretion rate 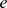 (tGSI = 22%, mGSI = 10%). Macrophage infection results in viral excretion and has a positive impact on the free viral particles, but it is attenuated by the virus mobilisation by infected macrophages.

The infection rate 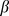 and the excretion rate 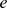 exhibited a strong interaction on the viral titer and the percentage of infected macrophages (GSI around 8%). Indeed, the viral replication needs macrophages to be infected and conversely, macrophage infection is induced by free viral particles which are released through viral excretion.

### Impact of host susceptibility and strain virulence on the infection resolution and associated immune mechanisms

The 37 scenarios corresponding to graduated levels of host susceptibility and strain virulence were simulated. The results are illustrated in Figure 5 and summarised in Table 4. The infection durations (52–118 days according to the scenario) were consistent with literature data [12], [16], [40], [41]. All scenarios had a notable impact on the infection duration. The scenarios related to macrophage permissiveness (S1
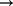
S2) induced higher differences in infection duration than the scenarios related to the cytokine synthesis (SB
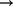
SA), even if the parameter variations were lower for scenarios S1
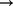
S2 than for scenarios SB
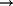
SA (Table 2). Consequently, the infection duration seems more sensitive to the parameters involved in the macrophage permissiveness than the antiviral cytokine synthesis rate.

**Table 4:** Summary of the virus and immune dynamics for variable host susceptibility and strain virulence.

	Susceptibility and virulence:				
	low		reference	high	
	S1	SB	S0	SA	S2
Virus – Infection duration [d]	52	57	72	93	118
Innate response – AUC					
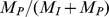 [%]	1.4	1.6	0.5	0.2	0.2
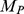	0.030	0.008	0.009	0.030	0.008
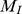	2.1	0.48	3.5	18	5.1
NK	71	15	225	866	559
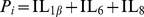	1.2	0.28	2.4	10.8	3.6
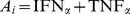	9	26	16	7	23
Adaptive response – AUC					
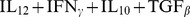	3.2	2.5	6.6	107	10.3
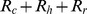	108	36	124	455	146
Cytokines – relative AUC [%]*					
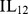	21	9	16	2.8	14
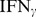	1	85	22	0.2	30
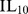	71	4.5	59	93	54
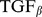	7	0.5	3	4	2
Orientation – relative AUC [%]*					
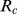	23	54	32	14	40
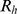	41	23	36	43	32
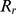	36	23	32	43	28

Scenarios S1: low macrophage permissiveness; SB: high antiviral and low immuno-modulatory cytokine synthesis; S0: reference; SA: high macrophage permissiveness; S2: low antiviral and high immuno-modulatory cytokine synthesis. AUC (area under the curve) units: macrophages [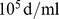], other cells [d/ml], cytokines [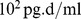]. Macrophages: infected (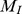), phagocyting (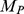). Adaptive effectors: cellular (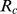), humoral (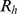) and regulatory (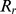) orientations.

*Relative AUC are defined within a group of outputs (*e.g.* the four cytokines 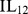, 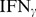, 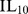 and 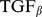) as the AUC of the outputs expressed as percentages of the sum of the AUC within the group.

The dynamics of immune components were similarly bell-shaped but differed quantitatively. More severe and longer infections were overall associated with higher levels of immune responses (Figure 5), but the relative proportions of the immune components varied (Table 4).

Concerning the innate response, we found a significant and positive correlation (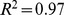) between the levels of infected macrophages and 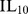, a cytokine which amplifies macrophage permissiveness and viral replication (results not shown).

There was no evidence of a link between the proportions of 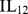 and 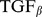 and the orientation of the adaptive response. The proportions of 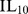 and 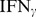, however, were linked to the adaptive response orientation (Table 4 & Figure 5). The proportion of 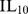 among 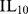 and 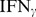 was negatively correlated with the percentage of cellular response (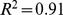) and positively correlated with both the humoral (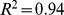) and regulatory responses (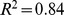).

Scenarios S1
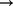
S2 resulted in immune dynamics rather close to the reference scenario, except for 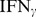 levels (Figure 5). On the one hand, high infection capacities (S0
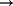
S2) resulted in long infection durations despite high levels of 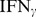 (Figure 5) and the adaptive response was oriented towards the cellular response (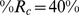, Table 4). However, 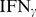 percentages were similar to the reference scenario. On the other hand, low infection capacities (S1
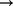
S0) resulted in short infection durations despite high percentages of 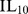 and the adaptive response was oriented towards the humoral response (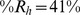,Table 4). 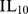 levels were similar to the reference scenario (Figure 5).

Scenarios SB
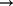
SA resulted in more contrasted immune dynamics (Figure 5) and influenced the adaptive response orientation more than scenarios S1
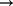
S2 (Table 4). Low antiviral capacities (S0
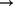
SA) resulted in long infection durations associated with high levels (Figure 5) and percentages of 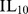, and co-dominant humoral and regulatory responses (Table 4). High antiviral capacities (SB
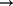
S0) resulted in short infection durations associated with high levels (Figue 5) and percentages of 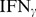, and an orientation towards the cellular response (Table 4).

**Figure 6:**
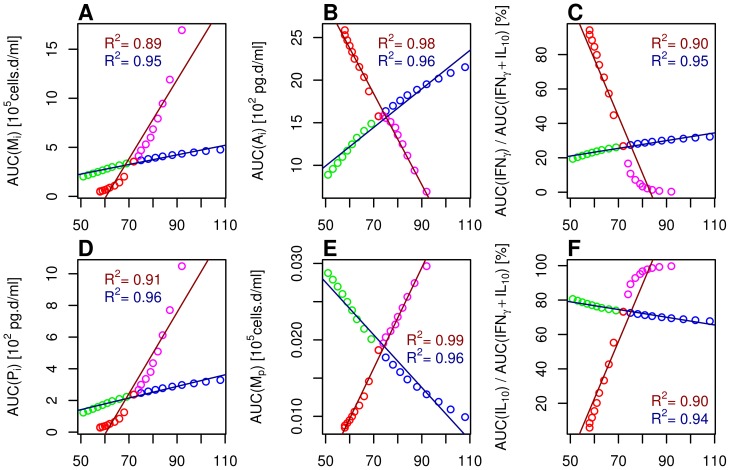
Linear regressions between the infection duration and immune components of interest. The immune components selected are the area under the curve (AUC) of **A**: infected macrophages (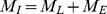), **B**: innate antiviral cytokines (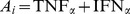), **D**: pro-inflammatory cytokines (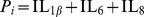), and **E**: phagocyting macrophages (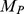); and the relative AUC of **C**: 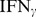 and **F**: 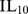. Two regressions were performed for each component: (i) for scenarios SB
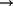
SA (dark red), in which the antiviral cytokine synthesis is higher (S0
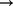
SB, red dots) or lower (S0
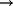
SA, magenta dots) than in the reference scenario; (ii) for scenarios S1
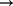
S2 (dark blue), in which the macrophage permissiveness is lower (S0
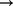
S1, green dots) or higher (S0
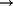
S2, blue dots) than in the reference scenario. Scenarios are defined in Table 2.

To extract trends more easily from these results, we investigated the correlations between the infection duration and the levels of seven key immune components of interest: infected and phagocyting macrophages, innate antiviral and pro-inflammatory cytokines and percentages of 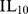 and 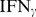 (Figure 6). Considering all scenarios together, no significant correlations could be extracted. Consequently, we split the scenarios in two groups: those with varying macrophage capacities (S1
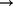
S2) and those with varying cytokine synthesis capacities (SB
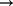
SA). All correlations were significant. The AUC of infected macrophages and pro-inflammatory cytokines were positively correlated with the infection duration for both groups. Otherwise, both groups exhibited opposite correlations.

In summary, low virulence and susceptibility scenarios induced short infection durations by promoting the phagocytosis or the synthesis of antiviral cytokines. On the contrary, high virulence and susceptibility scenarios resulted in long infection durations by promoting the infection and viral excretion or the synthesis of immuno-modulatory cytokines. Infection durations were always positively correlated with the levels of infected macrophages and pro-inflammatory cytokines. We observed that longer durations were associated with higher percentages of infected macrophages among activated macrophages. However, high levels of antiviral cytokines compared to immuno-regulatory cytokines, inducing a dominant cellular response, can be associated with either (i) long (scenarios related to macrophage permissiveness) or (ii) short infection durations (scenarios related to cytokine synthesis capacities).

## Discussion

### Modelling approach

In this paper, we presented an integrative dynamic model of the immune response in the lung to a virus targeting pulmonary macrophages: the PRRSv. The complexity level of the model is a good compromise between detailed intra-cellular models which focus on specific immune mechanisms and global models which give general trends [8]. Our model offers a comprehensive representation of the interactions between the virus and the immune response, which is necessary to explore the influence of the immune mechanisms on the infection duration. It is an original approach that takes into account the innate mechanisms, the adaptive response orientation and their complex interactions and regulations involving cytokines. We chose to represent the activation and orientation of the adaptive response, even if they occur outside the lung, because they interact with the immune and infection dynamics. Therefore, we did not detail the intermediate differentiation and proliferation steps of the adaptive response, but we represented its main immune functions and regulations. We hence obtained a realistic qualitative dynamic of the adaptive response. We did not represent the dendritic cells, major antigen presenting cells which influence the adaptive response activation and orientation. These cells maturate during their migration from the infection site to the lymph nodes, where they synthesise cytokines. They influence the infection dynamics through the cytokines they synthesise, which is consequently negligible in the lung. Moreover, dendritic cells and macrophages drive the adaptive response orientation in a similar way. As our model does intend to represent the orientation of the adaptive response between the different types and not the quantitative levels of adaptive cells, we trust that our simplification did not distort the results. This simplification is even more appropriate when dealing with PRRSv, as the virus also infects dendritic cells. Dendritic cells and macrophages hence have very similar dynamics and impacts during PRRSv infection [54], [55].

The model was built to describe a single infection by a stable pathogen at the within-host scale. We used it to study the impact of PRRSv strains, which exhibit various virulence levels. Our model could be easily adapted to other pathogens targeting pulmonary macrophages, such as influenza viruses. As influenza also infects epithelial cells, these target cells would have to be included in the model. As for other pathogens, the immune dynamic part of our model constitutes a good basis to study the innate response, given the fact that it is strongly simplify in most of the published models.

### Model calibration and scenario definition

The variation range of our model parameters were based on literature data. To complement these data and deal with the high variability on the parameter values and output levels, we developed an *ad hoc* method based on large parameter space exploration and sensitivity analysis. We defined a reference scenario, which corresponds to an average dynamics within the observed immune and infection dynamics. To study the impact of host and strain variability, we also defined parameter sets based on published assumptions and resulting in infection durations which were consistent with the literature [12], [16], [40], [41]. However, a quantitative calibration based on the viral dynamics and immune response data was not feasible. The levels of strain virulence and susceptibility of pigs are not quantified, the viral strains and pig breeds are not always informed and only few combinations of breeds and strains have been tested, so the comparisons between our scenarios and the literature are limited, especially for the immune response.

The sensitivity analysis highlighted five key parameters with a strong influence on the macrophage and virus dynamics: the viral inoculation dose 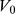, the viral excretion rate 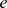, the macrophage infection rate 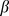, the phagocytosis rate 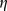 and the antiviral cytokine synthesis rate 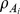. The inoculation dose is measured in experimental studies but is difficult to assess in field conditions. The other three key parameters are not easy to inform. Distinguishing between infected and phagocyting macrophages is an experimental challenge, so their dynamics are rarely observed and the related parameter values are not measured in the literature. Further experimentation would be needed to track the dynamics of our outputs of interest, especially viral titer and ideally both infected and phagocyting macrophages, or at least activated macrophages. The sensitivity analysis also exhibited high interactions between parameters, which partly explain the difficulties encountered to calibrate the model.

In terms of viral dynamics, the simulated infection durations ranged between 52 and 118 days according to the scenarios. Experimental studies show that the resolution generally occurs in the serum between 28 and 42 days after a PRRSv infection [12], [16], [40] and in the lung after 56 days on average [12], [56]–[59]. Infections longer than 240 days have been observed [16]. Consequently, the variation range of the simulated infection durations is realistic. Few studies measure the infection duration in the sera and in the lung simultaneously [40], [60], [61]. Combining these studies, we estimated that the infection duration in the lung is around 1.6 times longer than in the sera. This approximation allowed us to compare the infection duration in the lung from our simulation results to the infection duration in the blood (viremia) from experimental results. Few experimental studies focus on the response variability due to the viral strain or pig breed susceptibility. In a resistant pig breed, the viral load was around 35 days in the sera (estimated around 56 days in the lung) [52] and around 52 days in the lung with a low virulent strain [62]. Conversely, a more susceptible pig breed showed a 72-day viremia (estimated around 115 days in the lung) [52]. Infections by a highly virulent strain resulted in a viremia of 36 days (estimated around 58 days in the lung) [59] or the presence of viral particles in the lung for more than 67 days [63]. Our results were consistent with these data, but exhibited a larger range of infection durations.

In terms of immune response, the main trends found in the literature are the following: high virulence and susceptibility are associated with (i) a high activation of the immune response [64]; (ii) a dominant humoral response [38] with high levels of 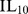; (iii) a lower cellular response with low levels antiviral cytokines [33], [34], [50], [64], [65]. However, trends (ii) and (iii) do not always hold. Some reviews point out that levels of antiviral and 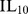 cytokines are highly variable between hosts and viral strains [11], [13]. An infection by a highly virulent strain can result in high levels of 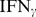 [59]. A strong cellular response is not necessarily correlated with a short infection duration [57]. Our results are qualitatively consistent with these data: high virulence and susceptibility scenarios were associated with high levels of the immune response and various orientations of the adaptive response. A common trend detected throughout all scenarios was the correlation between 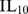 and the infected macrophages. Unlike the infected macrophages, 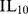 can be easily be measured. However, this result should be confirmed by experimentation before using 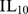 as a proxy for infected macrophages. We also found that high levels of pro-inflammatory cytokines were associated with longer infections. It has been suggested that inflammatory responses in the lung are an indicator of the severity and duration of the PRRSv infection rather than an indicator of the immune response efficacy [17].

### Assessing the impact of variability in host susceptibility and strain virulence

The strain virulence and pig susceptibility variability impact the infection duration, but the underlying mechanisms are still incompletely understood. Several hypotheses are formulated to explain PRRSv infection duration. Early immunological findings link prolonged viremia with (i) a weak innate antiviral response, (ii) high levels of immuno-modulatory cytokines (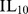 and 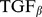) and (iii) low levels of 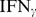, resulting in the orientation towards an inefficient humoral response; in contrast the cellular response could be protective. These results are challenged in more recent studies. All this knowledge is synthesised and discussed in terms of between-host and between-strain variability in recent reviews [16], [33], [34], [37]. In the following discussion sections, we confront our simulation results to the above-mentioned hypotheses.

#### Innate response

PRRSv has been reported to have various negative effects on innate immune functions, which probably contribute to the long survival of the virus in infected pigs. It suppresses the phagocyting activity, it fails to elicit any significant innate antiviral cytokines and it alters of the innate cytokine patterns compared to other respiratory pathogens [33], [37]. Consequently, we could expect negative correlations between the infection duration and both innate antiviral cytokines (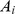) and phagocyting macrophages (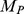). However, we found that long PRRSv infections were correlated as follows: either positively with 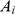 and negatively with 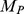, or positively with 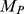 and negatively 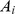. To explain these questioning results, we need to consider the levels of the other immune components and the parameter values used.

For scenarios S1
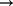
S2, we gradually promoted the infection and excretion while limiting the phagocytosis. It resulted in longer infection durations, a high increase of 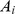, a decrease of 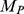 and a moderate increase of infected macrophages (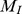). As 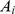 are mainly synthesised by 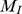, promoting infection results in increasing 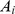. In turn, 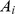 inhibits the infection and should reduce 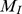. However, promoting the excretion and limiting the phagocytosis increase the free viral particles (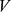) and 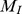. This last mechanism was dominant in these scenarios and countered the effect of 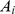.

For scenarios SB
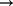
SA, we gradually promoted the synthesis of immuno-modulatory cytokines (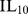 and 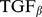) and limited the synthesis of 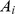 and 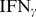. It resulted in longer infection durations, an increase of 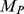 and a high increase of 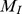 and 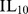. Promoting 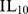 and 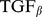 should increase the infection and reduce the phagocytosis, both contributing to an increase of 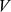. In turn, 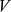 activates the phagocytosis and infection. This last mechanism was dominant in these scenarios and countered the cytokine effect. As a net result, 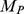 increased.

Our results suggest that despite high correlations between components of the innate response and the infection duration, measuring the innate response alone is insufficient to explain and predict the infection duration.

#### Adaptive response

The orientation towards the cellular, humoral or regulatory responses is supposed to have a high influence on the infection duration, but the mechanisms governing the orientation still need more insight. In experimental studies, the orientation towards the humoral and cellular responses is usually approximated by the levels of 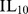 and 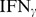 respectively. However, few studies consider the cellular and humoral responses simultaneously, as well as the associated cytokines, and most studies neglect the regulatory response. Reviews on PRRSv infection suggest that high levels of 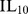 are capable of shifting the immune response towards a humoral response and that in the absence of 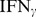, there is no cellular response [16], [33]. As the neutralisation of 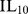 inhibits the regulatory response [37], levels of 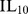 and regulatory response are assumed to be linked. In our model, the three orientations were represented, as well as their regulations and interactions. We found that the orientation of the adaptive response did not depend on specific cytokine levels, but on the proportions of 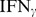 and 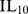. This result is consistent with the literature, as it points out the crucial role of 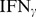 and 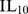 on the adaptive response orientation. However, it also points out the limits of the usual approximation of the adaptive response orientation by 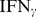 or 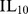 levels.

The cellular response is considered as protective against a wide variety of viral infections but its influence is controversial in the case of PRRSv infections [16], [33]. Reviews suggest that the suppression of 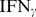 may have little influence on the *in vivo* disease progression [16], [66]. Moreover, long-term persistence of the virus in the host associated with a strong cellular response has been observed [33]. Both findings suggest that the cellular response alone cannot curtail the infection. Correlations between the strength of the cellular response and the PRRSv infection duration are highly variable between hosts and strains [34]. We also found that a dominant cellular response and high percentages of 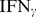 can be associated with either long or short infection durations. Scenarios SB
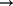
SA are consistent with the usual assumption that confers a protective role to the cellular response. However, in scenarios S1
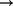
S2, long infection durations were associated with a dominant cellular response. To explain this result, we need to consider simultaneously the levels of the other immune components and the parameter values used. Long infection durations were associated with high levels of 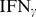 and 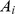, moderate levels of 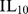 and infected macrophages, as well as an orientation towards the cellular response. We previously explained the high increase of 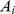 and the moderate increase of 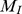 (see Innate response above). Being produced by 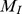, 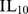 also increases, but less than 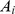 (lower production rate). As 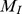 increases, the activation of the immune response also increases. In particular, the natural killers increase. They synthesise 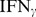, which promotes the cellular response, whose effectors synthesise 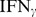, resulting in the orientation towards the cellular response. 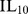 does not increase enough to prevent this orientation. As 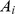, the cellular response and 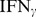 inhibit the infection, but not enough to compensate the high excretion and infection rates.

The high influence of the excretion rate on the infection duration is consistent with the results of the sensitivity analysis. The scenarios explored could correspond to real conditions. Indeed, an experimental study showed that pig genotypes can influence the alveolar macrophage abilityto suppress the viral replication [67]. Moreover, virulent strains vary in their ability to induce the synthesis of antiviral [16] and 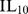 [37] cytokines. So scenarios S0
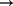
S2 could correspond to a pig that is not able to inhibit the viral replication and that is infected by a highly virulent type 2 PRRSv field strain, inducing a strong antiviral response and a moderate 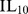 production.

Neutralising antibodies play a key role in the immunological control of a wide variety of viral infections [16], [33]. Consequently, a strong humoral response, should result in a short infection duration. PRRSv infections induce high levels of 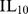 compared to the other cytokines and the humoral response levels are similar to the levels encountered in other viral infection. However, the levels of neutralising antibodies remain low. The combination of high levels of 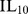 and a strong but inefficient humoral response is often proposed to explain the long infection duration [11]. Indeed, 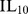 is a major regulator of the immune response and its inhibitory effects on numerous immune functions could explain several immunological phenomena observed in PRRSv infection [33], [34], [37]. However, the variability in host susceptibility and viral virulence challenges this hypothesis. PRRSv infections by virulent or attenuated strains showed no correlation between the 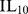 levels and the infection duration [16]. In a variety of studies, PRRSv infection resolution was observed without the development of neutralising antibodies [16]. We found that a dominant humoral response and high percentages of 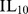 can be associated with either long or short infection durations. Scenarios SB
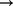
SA are consistent with the usual assumption of the ineffective humoral response. However, scenarios S1
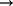
S0 associated short infection durations with a dominant humoral response. This result is due to the low excretion and macrophage infection rates, despite the low levels of innate and adaptive antiviral cytokines.

Concerning 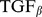 and the regulatory response, few studies explored their influences on the immune dynamics and the subsequent infection resolution. The induction of regulatory T lymphocytes (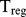) during the early stages of infection is considered as one of the mechanisms that establish chronic or persistent viral infections [16], [33]. According to this hypothesis, our results showed that a strong regulatory response was associated with very high levels of 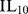 and that it resulted in a prolonged infection (scenarios S0
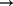
SA). Further experimentation considering the 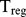 cells and 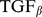 cytokines are needed to validate our model results.

## Conclusion

We built an original and integrative model of the immune response in the lung to a pathogen targeting pulmonary macrophages, applied here to PRRSv. This model provides an interesting framework to explore the macrophage–pathogen interactions while representing the adaptive response. We used the model to explore the influence of macrophage permissiveness and cytokine synthesis capacities on the infection duration and immune dynamics. A recent review suggests that the concepts proposed to explain prolonged PRRSv infection have not been experimentally proved; in particular, the roles of the cytokines and the orientation of the adaptive response need to be more clearly elucidated [16]. Our integrative model allowed to simulate contrasted dynamics in terms of immune response and infection duration, suggesting hypotheses to explain the apparent contradictions between published results.

In addition, we extracted some synthetic and original elements from our work.

1. Among the immune variables that can be easily measured, some were found to characterise immune mechanisms: (a) the proportions of 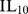 and 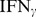 were good indicators of the adaptive response orientation; and (b) the level of 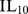 was a good indicator of the level of infected macrophages.
2. Whatever the strain virulence and host susceptibility, the infection duration was linked to some immune variables: (a) the level of pro-inflammatory cytokines was a good indicator of the infection duration; and (b) a dominant regulatory response was associated with a prolonged infection.

However, to identify and understand the immune mechanisms responsible for the infection duration, the entire immune response had to be considered. At least (i) the levels of innate antiviral cytokines, (ii) the level of 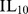, and (iii) the relative levels of 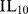 and 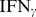 were needed.

We found that the macrophage permissiveness and the cytokine synthesis capacities both influence the infection duration through various immune mechanisms. Promoting antiviral cytokines or limiting the macrophage permissiveness and viral replication in order to reduce the infection duration has only been suggested [33], [34], [57]. Classically, two main approaches are associated to limit the infection: (i) appropriate housing conditions to reduce the pig susceptibility and (ii) vaccination to improve the immune response efficiency. Moreover, it has been shown that pig genotypes can influence the alveolar macrophage ability to suppress viral replication [51]. Our results suggest that the viral replication rate is highly influential on the infection duration. So selecting resistant pigs should be efficient to prevent severe infections. Concerning the vaccination strategies, vaccines capable of promoting the synthesis of antiviral cytokines or minimising 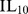 production have been considered in the literature and numerous experimentation have been carried out, but the current results are not convincing (reviewed in [16], [37]). Obviously, vaccination strategies need more insight. Our integrative model provides a powerful framework to go beyond experimental constraints. In particular, such an approach could be used to help designing efficient vaccination strategies.

## Supporting Information

Figure S1
**Preliminary sensitivity analysis: comparison of the phagocytosis and infection activities.** This figure results from the 243 simulations performed for the preliminary sensitivity analysis. **A**: Percentage of phagocyting macrophages among all macrophages over time (maximum 14%). **B**: Percentage of infected macrophages over time (maximum 100%). **C**: Phagocytosis activity as a percentage of the phagocytosis and infection flows, *i.e.* the ratio between the concentration of susceptible macrophages becoming phagocyting macrophages per unit of time and the concentration of susceptible macrophages becoming phagocyting or latent infected macrophages per unit of time 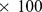. At a given time, if a simulation is above the 50% red line, its phagocytosis flow is higher than its infection flow. These figures show that, even if there are few phagocyting macrophages at all times, the phagocytosis activity can be dominant over the infection activity at given times for susceptible macrophages.(TIFF)

Figure S2
**Parameter space exploration: viral titer.** This figure results from the 6561 simulations performed for the sensitivity analysis. **A**: Viral titer over time (red curve: reference scenario S0). **B**: Distribution of the viral titer at day 200. Some simulations resulted in infection persistence, others in infection resolution occurring at various dates. The viral titer at day 200 was heterogeneously distributed: 56% of the simulations had a viral titer lower than 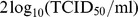, which is usually considered as the infection resolution; the remaining simulations had viral titers ranging between 2 and 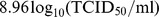. More precisely: (i) 3.7% of the simulations had a viral titer higher than the maximal initial inoculation titer (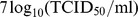) and (ii) 90% of the simulations had a viral titer lower than its corresponding inoculation titer (4, 5 or 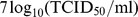). In the lung, PRRSv infection lasts 56 days on average [12] and can be longer than 200 days [16], [41].(TIFF)

Figure S3
**Parameter space exploration: cumulative number of phagocyting macrophages.** This figure results from the 6561 simulations performed for the sensitivity analysis. **A**: Cumulative number of phagocyting macrophages (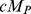) over time (red curve: reference scenario S0). **B**: Distribution of 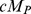 at day 1. **C**: Distribution of 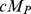 at day 200. 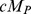 was highly variable between simulations: between 0.5 and 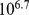 macrophages/ml on the first day, and between 1.4 and 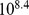 macrophages/ml at day 200. Most simulations rapidly increased during the first days and then tended to a threshold. This means that the phagocytosis activity was maximal at the beginning of the infection, which is consistent with the literature. Simulations that did not saturate corresponded to persistent infection. To our knowledge, there are no experimental studies that measure the concentration of phagocyting macrophages during a PRRSv infection.(TIFF)

Figure S4
**Parameter space exploration: percentage of infected macrophages.** This figure results from the 6561 simulations performed for the sensitivity analysis. **A**: Percentage of infected macrophages among all macrophages (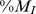) over time (red curve: reference scenario S0). **B**: Distribution of the 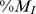 peak value. **C**: Distribution of the 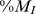 peak date. The peak is defined as the maximum value of 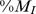 over the course of infection. The 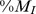 dynamics was highly variable among simulations but tended to decrease after the first weeks of infection. At day 200, 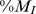 was higher than 60% for only 4% of the simulations and lower than 1% for 84% of the simulations. 55% of the simulations peaked during the first week. For 80% of the simulations, the 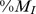 peak was lower than 20%. Some experimental studies showed a peak of infected macrophages of around 40% during the first week of a PRRSv infection [39]. During the first week, only 5% of the simulations had 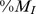 peaking between 20 and 60%, which is consistent with the experimental results.(TIFF)

File S1 (PDF)
