# Supplementary figures and images for: Integrative Model of the Immune Response to a Pulmonary Macrophage Infection: What Determines the Infection Duration?
Source: PLoS One. 2014 Sep 18;9(9):e107818. doi: 10.1371/journal.pone.0107818 (PMC4169448; doi:10.1371/journal.pone.0107818)

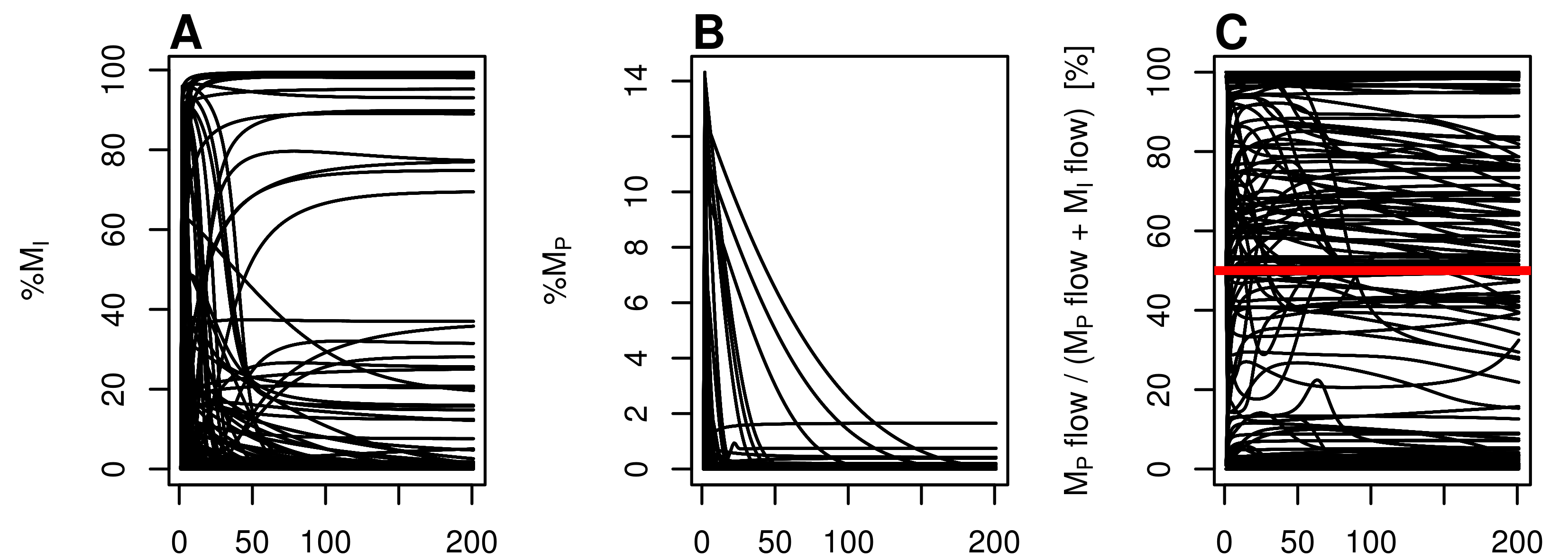

Supplement: Figure S1 — Preliminary sensitivity analysis: comparison of the phagocytosis and infection activities. This figure results from the 243 simulations performed for the preliminary sensitivity analysis. A: Percentage of phagocyting macrophages among all macrophages over time (maximum 14%). B: Percentage of infected macrophages over time (maximum 100%). C: Phagocytosis activity as a percentage of the phagocytosis and infection flows, i.e. the ratio between the concentration of susceptible macrophages becoming phagocyting macrophages per unit of time and the concentration of susceptible macrophages becoming phagocyting or latent infected macrophages per unit of time . At a given time, if a simulation is above the 50% red line, its phagocytosis flow is higher than its infection flow. These figures show that, even if there are few phagocyting macrophages at all times, the phagocytosis activity can be dominant over the infection activity at given times for susceptible macrophages. (TIFF) [file pone.0107818.s001.tiff]

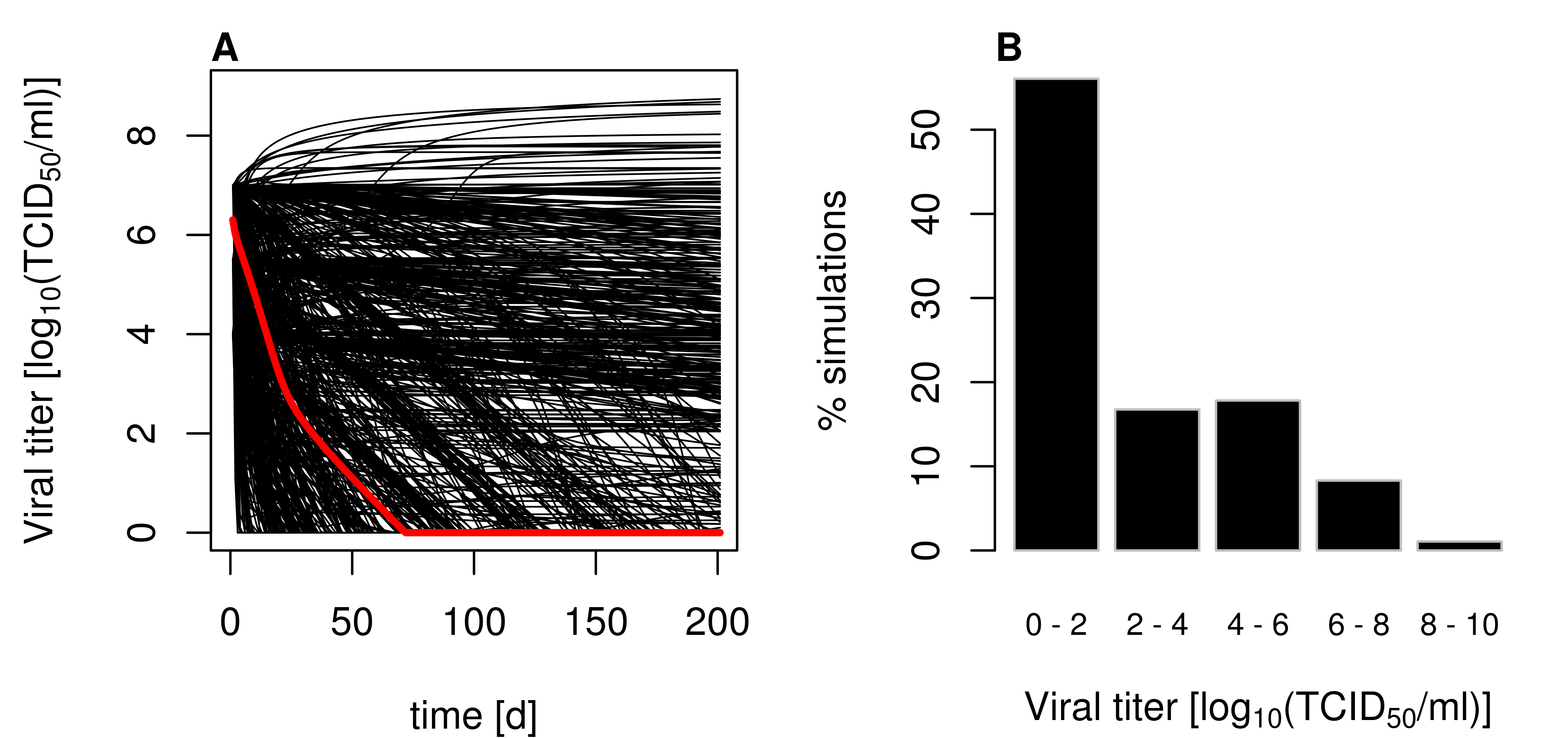

Supplement: Figure S2 — Parameter space exploration: viral titer. This figure results from the 6561 simulations performed for the sensitivity analysis. A: Viral titer over time (red curve: reference scenario S0). B: Distribution of the viral titer at day 200. Some simulations resulted in infection persistence, others in infection resolution occurring at various dates. The viral titer at day 200 was heterogeneously distributed: 56% of the simulations had a viral titer lower than , which is usually considered as the infection resolution; the remaining simulations had viral titers ranging between 2 and . More precisely: (i) 3.7% of the simulations had a viral titer higher than the maximal initial inoculation titer () and (ii) 90% of the simulations had a viral titer lower than its corresponding inoculation titer (4, 5 or ). In the lung, PRRSv infection lasts 56 days on average [12] and can be longer than 200 days [16], [41]. (TIFF) [file pone.0107818.s002.tiff]

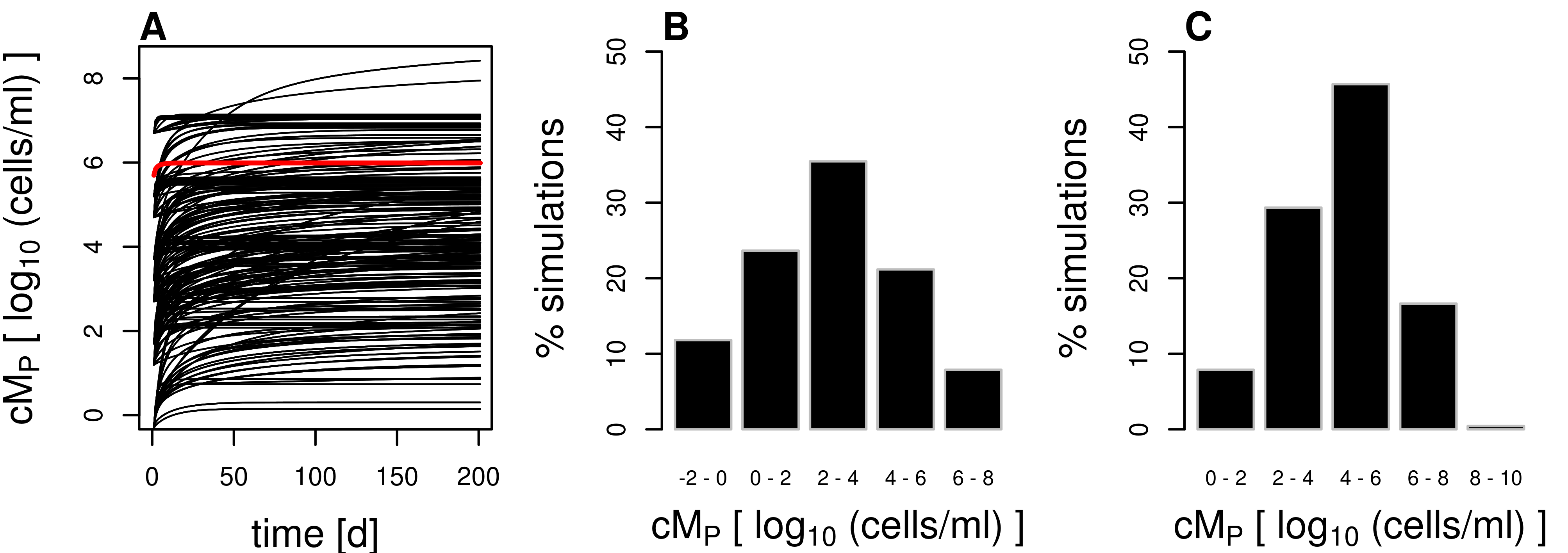

Supplement: Figure S3 — Parameter space exploration: cumulative number of phagocyting macrophages. This figure results from the 6561 simulations performed for the sensitivity analysis. A: Cumulative number of phagocyting macrophages () over time (red curve: reference scenario S0). B: Distribution of at day 1. C: Distribution of at day 200. was highly variable between simulations: between 0.5 and macrophages/ml on the first day, and between 1.4 and macrophages/ml at day 200. Most simulations rapidly increased during the first days and then tended to a threshold. This means that the phagocytosis activity was maximal at the beginning of the infection, which is consistent with the literature. Simulations that did not saturate corresponded to persistent infection. To our knowledge, there are no experimental studies that measure the concentration of phagocyting macrophages during a PRRSv infection. (TIFF) [file pone.0107818.s003.tiff]

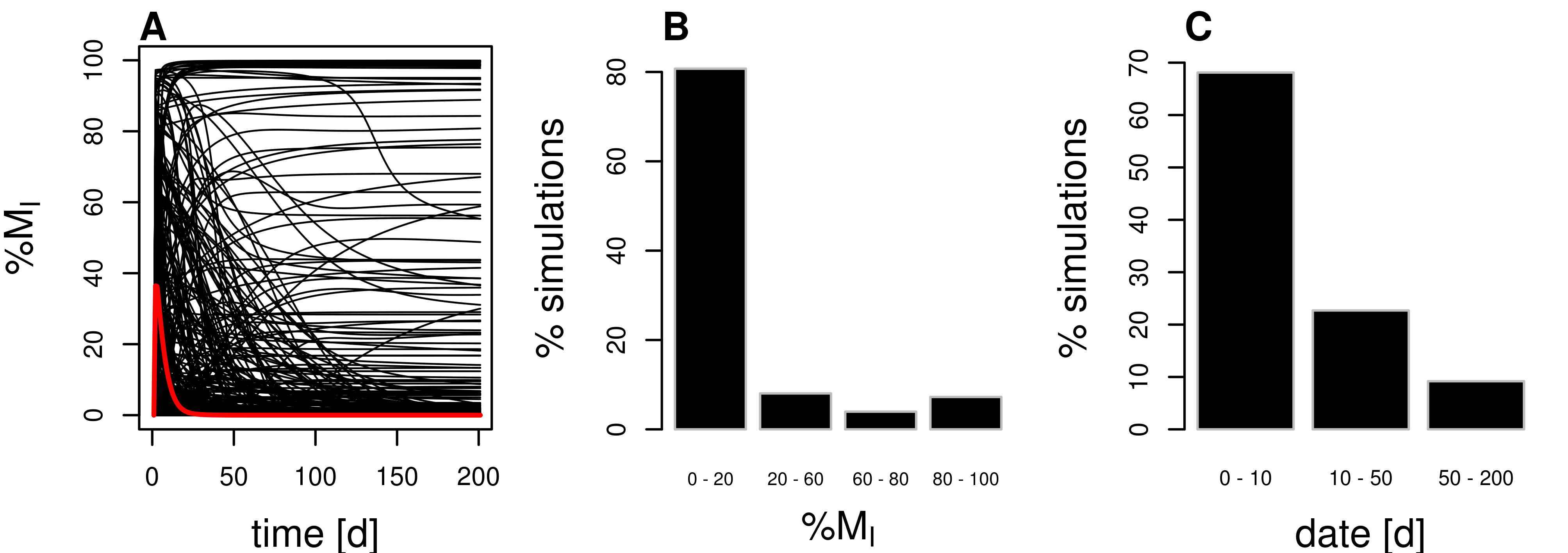

Supplement: Figure S4 — Parameter space exploration: percentage of infected macrophages. This figure results from the 6561 simulations performed for the sensitivity analysis. A: Percentage of infected macrophages among all macrophages () over time (red curve: reference scenario S0). B: Distribution of the peak value. C: Distribution of the peak date. The peak is defined as the maximum value of over the course of infection. The dynamics was highly variable among simulations but tended to decrease after the first weeks of infection. At day 200, was higher than 60% for only 4% of the simulations and lower than 1% for 84% of the simulations. 55% of the simulations peaked during the first week. For 80% of the simulations, the peak was lower than 20%. Some experimental studies showed a peak of infected macrophages of around 40% during the first week of a PRRSv infection [39]. During the first week, only 5% of the simulations had peaking between 20 and 60%, which is consistent with the experimental results. (TIFF) [file pone.0107818.s004.tiff]
