# Supplementary material for: Integrative Model of the Immune Response to a Pulmonary Macrophage Infection: What Determines the Infection Duration?
Source: PLoS One. 2014 Sep 18;9(9):e107818. doi: 10.1371/journal.pone.0107818 (PMC4169448; doi:10.1371/journal.pone.0107818)
Supplement: File S1 — (PDF) [file pone.0107818.s005.pdf]

Supporting Information to
Integrative Model of the Immune Response to a Pulmonary Macrophage Infection: What Determines the Infection Duration?

Natacha Go[1,2*], Caroline Bidot[1], Catherine Belloc[2], Suzanne Touzeau[3,4]

1 UR341 MIA, INRA, Jouy-en-Josas, France
2 LUNAM Université, Oniris, INRA UMR 1300 BioEpAR, Nantes, France
3 UMR1355 ISA, INRA, Université Nice Sophia Antipolis, CNRS, Sophia Antipolis, France
4 BIOCORE, Inria, Sophia Antipolis, France
∗ E-mail: Natacha.Go@jouy.inra.fr

S1. Complete model description

This model describes the infection and immune dynamics induced by a pathogen targeting pulmonary macrophages in the lung. It focuses on the macrophage–virus interactions and highly details the innate immune response and cytokine regulations. The adaptive immune response is less detailed but includes the cellular, humoral and regulatory orientations and their main functions.

The model is characterised by 18 state variables: the free viral particles; five effectors of the innate response, consisting of four macrophage states (susceptible, phagocyting, infected and latent, infected and excreting the virus) and the natural killers; three effectors of the adaptive response, representing the cellular, humoral and regulatory responses; nine cytokines composed of the major pro-inflammatory ($IL_{1\beta}$, IL_6, IL_8), the innate antiviral (TNF_α, IFN_α) and the immuno-regulatory (IFN_γ, IL_{12}, IL_{10}, TGF_β) cytokines.

We chose a deterministic continuous-time dynamic framework. Our model is hence a set of 18 ordinary differential equations, which represent the evolution over time of the state variables. The main processes that drive their evolution over time and that are integrated in the model are: the phagocytosis of the viral particles by the macrophages; the macrophage infection by the virus; the excretion of free viral particles by the infected macrophages; the recruitment and decay/migration of the macrophages; the activation and decay/migration of the other effectors; the cytokine productions by the immune cells and their decay; the cytokine regulations.

The functional diagram of the system appears in FIGURE 1 (main text). FIGURE 2 (main text) gives a schematic representation of the model (without regulations). Parameter descriptions and values are synthesised in TABLE 1 (main text). The cytokine regulations and syntheses represented in our model as well as the related literature references are summarised in TABLE S1 and TABLE S2 respectively.

We first present how the cytokine regulations are formalised in the model and then describe the dynamics of the various state variables: macrophages, free viral particles, natural killers, effectors of the adaptive response and cytokines.

Cytokine regulations

Cytokines regulate the cellular functions through their recognition by specific receptors, inducing cascaded reactions within the cells. The higher the cytokine concentration, the stronger the effect. However,

there is a limited number of cytokine receptors on the cell surface, so the effect saturates above a given cytokine concentration. We formalised the cytokine effects by a Michaelis–Menten function (κ) of the cytokine concentration (C_i) as follows:

$$\kappa(C_i) = \frac{K\,C_i}{C_i + k},$$

where K represents the saturation factor and k the half-saturation cytokine concentration [1–3].

Considering a flow (R), which can either be an inflow (*e.g.* recruitment) or an outflow (*e.g.* decay), a cytokine can have three possible effects listed below.

- Activation: $R\ \kappa(C_i)$. The flow is only possible in the presence of the cytokine and it increases with the cytokine concentration.
- Amplification: $R\ [1+\kappa(C_i)]$. The flow increases with the cytokine concentration.
- Inhibition: $R/[1+\kappa(C_i)]$. The flow decreases with the cytokine concentration.

Regulations often involve several cytokines (C_i and C_j), which can act

- either independently:
  - co-activation: $R\ [\kappa(C_i)+\kappa(C_j)]$,
  - co-amplification: $R\ [1+\kappa(C_i)]\ [1+\kappa(C_j)]$,
  - co-inhibition: $R/([1+\kappa(C_i)]\ [1+\kappa(C_j)])$;
- or in synergy:
  - co-activation: $R\ \kappa(C_i,C_j) = R\ \kappa(C_i)\ \kappa(C_j)$,
  - co-amplification: $R\ [1+\kappa(C_i,C_j)] = R\ [1+\kappa(C_i)\ \kappa(C_j)]$,
  - co-inhibition:$R/[1+\kappa(C_i,C_j)] = R/[1+\kappa(C_i)\ \kappa(C_j)]$.

As very few studies estimate the regulation parameters (k and K) in the literature [3], we used the same parameter values for all cytokine regulations.

Macrophage dynamics

Macrophages phagocyte and destroy the virus, but they are also target cells for the virus. Activated macrophages (either phagocyting or infected) present the viral antigen and activate the adaptive response. The macrophage activation also induces the synthesis of innate cytokines: pro-inflammatory ($\mathrm{IL}_{1\beta}, \mathrm{IL}_6, \mathrm{IL}_8$), antiviral ($\mathrm{IFN}_\alpha, \mathrm{TNF}_\alpha$) and immuno-regulatory ($\mathrm{IL}_{12}, \mathrm{IL}_{10}, \mathrm{TGF}_\beta$) cytokines [4].

States In the model, macrophages can either be susceptible (M_S), phagocyting (M_P), or infected; in this latter case, they are either latent (M_L) or excreting the virus (M_E). We represented the evolution over time of the macrophage concentrations for these four states.

Decay All macrophage states are submitted to natural death or/and migration (rate μ_M^{nat}), as well as apoptosis induced by TNF_α (rate μ_M^{ap}) [5]. The natural death rate is considered higher for infected macrophages than for susceptible and phagocyting macrophages (multiplicative factor δ_μ). Moreover, infected macrophages can be destroyed by natural killers (rate μ_M^{inn}) and cells from the cellular response (rate μ_M^{ad}).

Recruitment Susceptible macrophages are recruited from the bloodstream (rate A_m). Cytokines IL_6 and IL_{12} co-amplify the macrophage recruitment [6–8] in synergy and IL_8 attracts the macrophages in the infection place [8]. In the absence of virus, the cytokine concentrations are supposed to be negligible and the resulting concentration of susceptible macrophages in the lung is constant: $M_S = M_S^0 = \frac{A_m}{\mu_M^{\mathrm{nat}}}$.

Phagocytosis and infection When susceptible macrophages encounter free viral particles (V), they can either phagocyte the virus (rate η) or become infected (rate β). We assume that phagocyting macrophages revert to the susceptible state after viral destruction (rate γ), whereas infected macrophages remain infected (*i.e.* they cannot eliminate the virus). We also assume that the infected and phagocyting states are exclusive and that once phagocyting or infected, macrophages cannot phagocyte or be infected by other viral particles [9].

The phagocytosis is amplified by the antiviral cytokines ($\mathrm{IFN}_\alpha, \mathrm{TNF}_\alpha, \mathrm{IFN}_\gamma$) and inhibited by the immuno-modulatory cytokines ($\mathrm{IL}_{10}, \mathrm{TGF}_\beta$) – . The phagocytosis ending is amplified by the antiviral cytokines and inhibited by IL_{10}. The macrophage infection is amplified by IL_{10} and inhibited by innate antiviral cytokines ($\mathrm{IFN}_\alpha, \mathrm{TNF}_\alpha$) and TGF_β.

Viral excretion Infected macrophages are first latent (mean duration $1/\lambda$) before they start excreting. The transition between the latent and excreting states (rate λ) is inhibited by the antiviral cytokines. Excretion is supposed to be transitory and excreting macrophages may revert to the latent state in the presence of antiviral cytokines.

$$
\begin{aligned}
\dot{M}_S = {} & A_m\ [1+\kappa(\mathrm{IL}_{12}, \mathrm{IL}_6)]\ [1+\kappa(\mathrm{IL}_8)] && \longleftarrow \textsf{recruitment} \\
& - \eta\ M_S\ V\ \frac{[1+\kappa(\mathrm{TNF}_\alpha)]\ [1+\kappa(\mathrm{IFN}_\alpha)]\ [1+\kappa(\mathrm{IFN}_\gamma)]}{[1+\kappa(\mathrm{IL}_{10})]\ [1+\ \kappa(\mathrm{TGF}_\beta)]} && \longleftarrow \textsf{phagocytosis} \\
& + \gamma\ M_P\ \frac{[1+\kappa(\mathrm{TNF}_\alpha)]\ [1+\kappa(\mathrm{IFN}_\alpha)]\ [1+\kappa(\mathrm{IFN}_\gamma)]}{1+\kappa(\mathrm{IL}_{10})} && \longleftarrow \textsf{phagocytosis ending} \\
& - \beta\ M_S\ V\ \frac{1+\kappa(\mathrm{IL}_{10})}{[1+\kappa(\mathrm{TNF}_\alpha)]\ [1+\kappa(\mathrm{IFN}_\alpha)]\ [1+\kappa(\mathrm{TGF}_\beta)]} && \longleftarrow \textsf{infection} \\
& - M_S\ (\mu_M^{\mathrm{nat}} + \mu_M^{\mathrm{inf}}\ \mathrm{TNF}_\alpha) && \longleftarrow \textsf{decay}
\end{aligned}
$$

$$
\begin{aligned}
\dot{M}_P = {} & \eta\ M_S\ V\ \frac{[1+\kappa(\mathrm{TNF}_\alpha)]\ [1+\kappa(\mathrm{IFN}_\alpha)]\ [1+\kappa(\mathrm{IFN}_\gamma)]}{[1+\kappa(\mathrm{IL}_{10})]\ [1+\ \kappa(\mathrm{TGF}_\beta)]} && \longleftarrow \textsf{phagocytosis} \\
& - \gamma\ M_P\ \frac{[1+\kappa(\mathrm{TNF}_\alpha)]\ [1+\kappa(\mathrm{IFN}_\alpha)]\ [1+\kappa(\mathrm{IFN}_\gamma)]}{1+\kappa(\mathrm{IL}_{10})} && \longleftarrow \textsf{phagocytosis ending} \\
& - M_P\ (\mu_M^{\mathrm{nat}} + \mu_M^{\mathrm{ap}}\ \mathrm{TNF}_\alpha) && \longleftarrow \textsf{decay}
\end{aligned}
$$

$$
\begin{aligned}
\dot{M}_L = {} & \beta\ M_S\ V\ \frac{1+\kappa(\mathrm{IL}_{10})}{[1+\kappa(\mathrm{TNF}_\alpha)]\ [1+\kappa(\mathrm{IFN}_\alpha)]\ [1+\kappa(\mathrm{TGF}_\beta)]} && \longleftarrow \textsf{infection} \\
& - \lambda\ M_L\ \frac{1}{[1+\kappa(\mathrm{TNF}_\alpha)]\ [1+\kappa(\mathrm{IFN}_\gamma)]\ [1+\kappa(\mathrm{IFN}_\alpha)]} && \longleftarrow \textsf{excretion} \\
& + \nu\ M_E\ [\kappa(\mathrm{TNF}_\alpha) + \kappa(\mathrm{IFN}_\gamma) + \kappa(\mathrm{IFN}_\alpha)] && \longleftarrow \textsf{excretion ending} \\
& - M_L\ (\mu_M^{\mathrm{nat}}\ \delta_\mu + \mu_M^{\mathrm{ap}}\ \mathrm{TNF}_\alpha + \mu_M^{\mathrm{inn}}\ \mathrm{NK} + \mu_M^{\mathrm{ad}}\ R_c) && \longleftarrow \textsf{decay}
\end{aligned}
$$

$$
\begin{aligned}
\dot{M}_E = {} & \lambda\ M_L\ \frac{1}{[1+\kappa(\mathrm{TNF}_\alpha)]\ [1+\kappa(\mathrm{IFN}_\gamma)]\ [1+\kappa(\mathrm{IFN}_\alpha)]} && \longleftarrow \textsf{excretion} \\
& - \nu\ M_E\ [\kappa(\mathrm{TNF}_\alpha) + \kappa(\mathrm{IFN}_\gamma) + \kappa(\mathrm{IFN}_\alpha)] && \longleftarrow \textsf{excretion ending} \\
& - M_E\ (\mu_M^{\mathrm{nat}}\ \delta_\mu + \mu_M^{\mathrm{ap}}\ \mathrm{TNF}_\alpha + \mu_M^{\mathrm{inn}}\ \mathrm{NK} + \mu_M^{\mathrm{ad}}\ R_c) && \longleftarrow \textsf{decay}
\end{aligned}
$$

Viral dynamics

The virus enters the body through the mucosal surfaces of the respiratory tract and replicates in the pulmonary macrophages.

We represented the evolution over time of the free viral particles (V). When they encounter susceptible macrophages (M_S), they can either be phagocyted by the macrophages (rate η), resulting in viral destruction, or they can infect the macrophages (rate β), resulting in virus replication. Free viral particles are released in the lung by excreting macrophages (rate e). They can be neutralised by antibodies represented by the humoral response R_h (rate μ_V^{ad}). They are also submitted to natural death and migration outside the lung (rate μ_V^{nat}).

$$\begin{aligned}
\dot{V} = {} & e\ M_E && \longleftarrow \textsf{replication}\\
& -\eta\ M_S\ V\ \frac{[1+\kappa(\text{TNF}_\alpha)]\ [1+\kappa(\text{IFN}_\alpha)]\ [1+\kappa(\text{IFN}_\gamma)]}{[1+\kappa(\text{IL}_{10})]\ [1+\ \kappa(\text{TGF}_\beta)]} && \longleftarrow \textsf{phagocytosis}\\
& -\beta\ M_S\ V\ \frac{1+\kappa(\text{IL}_{10})}{[1+\kappa(\text{TNF}_\alpha)]\ [1+\kappa(\text{IFN}_\alpha)]\ [1+\kappa(\text{TGF}_\beta)]} && \longleftarrow \textsf{infection}\\
& -V\ (\mu_V^{\text{nat}}+\mu_V^{\text{ad}}\ R_h) && \longleftarrow \textsf{decay}
\end{aligned}$$

Natural killer dynamics

Natural killers are effectors of the innate response. Their main immune functions are the destruction of infected cells and IFN_γ synthesis [4, 8, 10, 11]. These cytotoxic cells are recruited on the infection by pro-inflammatory cytokines. Their proliferation and immune functions are activated by several cytokines ($\text{IFN}_\gamma, \text{IL}_{12}, \text{IL}_{15}, \text{IL}_{18}, \text{IL}_{21}, \text{IFN}_{\alpha\beta}$), whereas IL_{10} inhibits the natural killer differentiation and their immune functions [10].

We represented the dynamics of activated natural killers (NK) and only included the regulations by the most influential cytokines. The recruitment of natural killers from the bloodstream (rate α_N) requires pro-inflammatory cytokines: IL_{12} and IL_6 co-activate the recruitment, whereas IL_8 acts independently. Natural killers are then activated by IFN_γ and IL_{12}, whereas IL_{10} inhibits the activation. They are submitted to natural death or/and migration (rate μ_R).

$$\begin{aligned}
\dot{\text{NK}} = {} & \alpha_N\ \frac{[\kappa(\text{IL}_{12}, \text{IL}_6)+\kappa(\text{IL}_8)]\ [\kappa(\text{IFN}_\gamma)+\kappa(\text{IL}_{12})]}{[1+\kappa(\text{IL}_{10})]} && \longleftarrow \textsf{recruitment \& activation}\\
& -\mu_R\ \text{NK} && \longleftarrow \textsf{decay}
\end{aligned}$$

Adaptive effector dynamics

The adaptive response activation begins with the naive CD_4^+ T cell differentiation by antigen presenting cells (dendritic cells or macrophages) within the lymphoid tissues. Depending on the cytokine profiles in the lymph nodes, CD_4^+ T cells differentiate in one of three main CD_4^+ effector types, which each have specific immune functions: type 1 T helpers (T_{h1}, belonging to the cellular response), type 2 T helpers (T_{h2}, belonging to the humoral response) and regulatory T lymphocytes (T_{reg}, belonging to the regulatory response) [4, 12–19]. The differentiation towards T_{h1} is induced by IL_{12} and amplified by IFN_γ. The differentiation towards T_{h2} is induced by IL_6 and amplified by IL_4. The differentiation towards T_{reg} is induced by TGF_β and amplified by IL_{10}. The cytokines responsible for the differentiation towards a CD_4^+ effector type simultaneously inhibit the differentiation towards the two other types. After the differentiation step, the CD_4^+ effectors express their specific immune functions and proliferate. T_{h1} synthesise the IL_2 and IFN_γ cytokines. In addition, they are responsible for the differentiation of the CD_8^+ T cells in cytotoxic lymphocytes (CTL). CTL synthesise IFN_γ and TNF_α and destroy the infected

macrophages. T_{h2} synthesise IL_4 and IL_{10}. In addition, they are the main activator of the B lymphocytes (through IL_4 and IL_{10}), which produce the neutralising antibodies. T_{reg} synthesise the TGF_β and IL_{10} immuno-modulatory cytokines. In summary:

- The cellular response: (i) includes the T_{h1} and CTL; (ii) is activated by IL_{12} and IFN_γ and inhibited by IL_6, IL_{10} and TGF_β; and (iii) is responsible for the synthesis of IL_2, IFN_γ and TNF_α.
- The humoral response: (i) includes the T_{h2}, B lymphocytes and antibodies; (ii) is activated by IL_6, IL_4 and IL_{10} and inhibited by IL_{12}, IFN_γ and TGF_β; and (iii) is responsible for the synthesis of IL_4 and IL_{10} and for the viral particle neutralisation.
- The regulatory response: (i) only includes the T_{reg}; (ii) is activated by TGF_β and IL_{10} and inhibited by IL_6, IL_{12} and IFN_γ; and (iii) is responsible for the immune response inhibition through its synthesis of the TGF_β and IL_{10} immuno-modulatory cytokines.

In our model, we represented the adaptive response by three effectors corresponding to the three main orientations: cellular (R_c), humoral (R_h) and regulatory (R_r) responses. As for the NK cells, we only represented the dynamics of the activated effectors.

Based on the model proposed by Yates *et al.* for the regulation of T helper cell populations [19], we synthesised the dynamics of each adaptive effector by three steps: activation by activated macrophages (rate α_R), proliferation (rate p_R) and decay. We represented the regulations of the activation and proliferation steps by the most influential cytokines: IFN_γ, IL_{12}, IL_{10} and TGF_β.

The decay includes the natural decay (rate μ_R) and the Activation Induced Cell Death (AICD) induced by the interaction with a T_{h1} from the R_c compartment (rate δ_{R_c}) [19].

Cellular response [16–18, 20–23] R_c represents the type 1 T helper cells and the cytotoxic lymphocytes. Activation is amplified by IFN_γ and IL_{12} and inhibited by IL_{10}. Proliferation is activated by IFN_γ and IL_{12} and inhibited by IL_{10} and TGF_β. R_c synthesises IFN_γ and is responsible for the destruction of infected cells.

$$\begin{aligned}
\dot{R_c} &= \alpha_R\,(M_P + M_L + M_E)\,\frac{[1+\kappa(\mathrm{IFN}_\gamma)]\,[1+\kappa(\mathrm{IL}_{12})]}{1+\kappa(\mathrm{IL}_{10})} && \longleftarrow \text{activation}\\
&+ p_R\,R_c\,\frac{[\kappa(\mathrm{IFN}_\gamma)+\kappa(\mathrm{IL}_{12})]}{[1+\kappa(\mathrm{IL}_{10})]\,[1+\kappa(\mathrm{TGF}_\beta)]} && \longleftarrow \text{proliferation}\\
&- \mu_R\,R_c - \delta_{R_c}\,{R_c}^2 && \longleftarrow \text{decay}
\end{aligned}$$

Humoral response [16–18, 24] R_h represents the type 2 T helper cells, the B lymphocytes and the antibodies. Activation is amplified by IL_{10} and inhibited by IFN_γ and IL_{12}. Proliferation is activated by IL_{10} and inhibited by IFN_γ, IL_{12} and TGF_β. R_h synthesises IL_{10} and is responsible for the neutralisation of free viral particles through antibodies.

$$\begin{aligned}
\dot{R_h} &= \alpha_R\,(M_P + M_L + M_E)\,\frac{[1+\kappa(\mathrm{IL}_{10})]}{[1+\kappa(\mathrm{IFN}_\gamma)]\,[1+\kappa(\mathrm{IL}_{12})]} && \longleftarrow \text{activation}\\
&+ p_R\,R_h\,\frac{\kappa(\mathrm{IL}_{10})}{[1+\kappa(\mathrm{IFN}_\gamma)]\,[1+\kappa(\mathrm{IL}_{12})]\,[1+\kappa(\mathrm{TGF}_\beta)]} && \longleftarrow \text{proliferation}\\
&- \mu_R\,R_h - \delta_{R_c}\,R_c\,R_h && \longleftarrow \text{decay}
\end{aligned}$$

Regulatory response [16–18, 25] R_r represents the represents the regulatory T cells. Activation is amplified by IL_{10} and TGF_β and inhibited by IFN_γ and IL_{12}. Proliferation is activated by TGF_β and inhibited by IL_{10}, IFN_γ and IL_{12}. R_r synthesises IL_{10} and TGF_β.

$$\begin{aligned}
\dot{R_r} &= \alpha_R \, (M_P + M_L + M_E) \, \frac{[1+\kappa(\mathrm{IL}_{10})] \, [1+\kappa(\mathrm{TGF}_\beta)]}{[1+\kappa(\mathrm{IFN}_\gamma)] \, [1+\kappa(\mathrm{IL}_{12})]} && \longleftarrow \text{activation} \\
&+ p_R \, R_r \, \frac{\kappa(\mathrm{TGF}_\beta)}{[1+\kappa(\mathrm{IL}_{10})] \, [1+\kappa(\mathrm{IFN}_\gamma)] \, [1+\kappa(\mathrm{IL}_{12})]} && \longleftarrow \text{proliferation} \\
&- \mu_R \, R_r - \delta_{R_c} \, R_c \, R_r && \longleftarrow \text{decay}
\end{aligned}$$

Cytokine dynamics

Cytokines are small proteins that play a key role in cell-signalling. They are produced by activated immune cells and affect the behaviour of other cells, sometimes the releasing cell itself. They act through specific membranous receptors inducing cascaded reactions within the target cell. They have various functions. In particular, cytokines modulate the balance between the humoral and cellular responses. Some cytokines enhance or inhibit the action of other cytokines in complex ways.

In the model, we only integrated the regulations by the nine most influential cytokines. We grouped them into three classes depending on their main function: pro-inflammatory ($IL_{1\beta}, IL_6, IL_8$), antiviral ($TNF_\alpha, IFN_\alpha, IFN_\gamma$) and immuno-regulatory ($IL_{12}, IL_{10}, TGF_\beta$) cytokines. TNF_α is generally considered as a pro-inflammatory cytokine, but we were here more interested is its antiviral function.

We assumed that the cytokines are efficient enough in order to neglect their consumption when they interact with a cell. So the cytokine dynamics results from their synthesis by immune cells (rates ρ_x, where $_x$ depends on the cytokine considered) and their natural death (rate μ_C)

The main cytokine regulations are summarised in Table S1 and the cytokine syntheses in Table S2.

Pro-inflammatory cytokines [4, 6–8, 31, 35] Pro-inflammatory cytokines $IL_{1\beta}$, IL_6 and IL_8 amplify the recruitment of macrophages and activated natural killers. They are synthesised by the activated macrophages. As we had no information on their respective production rate, we used the same synthesis rate (ρ_{P_i}) for the three cytokines. The synthesis of $IL_{1\beta}$ is inhibited by IL_{10}, whereas the synthesis of IL_6 and IL_8 is co-activated by $IL_{1\beta}$ and TNF_α.

$$\begin{aligned}
\dot{\mathrm{IL}_{1\beta}} &= \rho_{P_i} \, (M_P + M_L + M_E) \, \frac{1}{1+\kappa(\mathrm{IL}_{10})} && \longleftarrow \text{synthesis} \\
&- \mu_C \, \mathrm{IL}_{1\beta} && \longleftarrow \text{decay}
\end{aligned}$$

$$\begin{aligned}
\dot{\mathrm{IL}_6} &= \rho_{P_i} \, (M_P + M_L + M_E) \, \kappa(\mathrm{IL}_{1\beta}, \mathrm{TNF}_\alpha) && \longleftarrow \text{synthesis} \\
&- \mu_C \, \mathrm{IL}_6 && \longleftarrow \text{decay}
\end{aligned}$$

$$\begin{aligned}
\dot{\mathrm{IL}_8} &= \rho_{P_i} \, (M_P + M_L + M_E) \, \kappa(\mathrm{IL}_{1\beta}, \mathrm{TNF}_\alpha) && \longleftarrow \text{synthesis} \\
&- \mu_C \, \mathrm{IL}_8 && \longleftarrow \text{decay}
\end{aligned}$$

Antiviral cytokines Antiviral cytokines promote the phagocytosis and reduce the infection by inhibiting the macrophage permissiveness and/or the viral replication.

The innate antiviral cytokines TNF_α and IFN_α are synthesised by activated macrophages [5, 6, 20, 21, 26–29, 31]. IL_{10} inhibits the synthesis of TNF_α.

Table S1. Cytokine regulations.

	Cytokines						
	**pro-inf.**	**innate antiviral**		**immuno-regulatory**			
	P_i*	TNF_{α}	IFN_{α}	IFN_{γ}	IL_{12}	IL_{10}	TGF_{β}
Innate response							
Macrophage recruitment	+				+		
NK recruitment & activation	+			+	+	−	
Macrophage apoptosis		+					
Phagocytosis		+	+	+		−	−
Macrophage permissiveness		−	−	−		+	−
Viral replication		−	−	−			
Adaptive response							
Cellular response				+	+	−	−
Humoral response				−	−	+	−
Regulatory response				−	−	±	+
Cytokine syntheses							
Pro-inflammatory $IL_{1\beta}$						−	
IL_6, IL_8	+	+					
Innate antiviral TNF_{α}						−	
Adaptive antiviral IFN_{γ}		+	+	+	+	−	−
Immuno-regulatory IL_{12}						−	
IL_{10}						+	±
Specific references	[7, 8, 23]	[5, 20, 21, 26–29]		[20–23]	[24]	[7, 30–35]	[25]
Common references		[6, 31]				[16–18]	
Global references				[4, 12–15, 36–38]			

Main positive (+) and negative (−) regulations included in the model for various immune mechanisms.
* Pro-inflammatory cytokines $P_i = IL_{1\beta}, IL_6, IL_8$

Table S2. Cytokine syntheses.

	Cytokines							
	**pro-inf.**	**innate antiviral**		**immuno-regulatory**				
	P_i*	TNF_{α}	IFN_{α}	IFN_{γ}	IL_{12}	IL_{10}	TGF_{β}	References
Innate cells								
Activated macrophages	✓	✓	✓		✓	✓		[8, 39]†
Activated natural killers				✓				[8, 11]†
Adaptive cells								
Cellular effectors				✓				†
Humoral effectors						✓		†
Regulatory effectors						✓	✓	†

Production of cytokines included (✓) in the model by innate or adaptive immune cells.
* Pro-inflammatory cytokines $P_i = IL_{1\beta}, IL_6, IL_8$
† Global references: [4, 12–15, 17, 36, 40, 41]

$$\dot{\text{TNF}}_\alpha = \rho_{\text{TNF}_\alpha}\,(M_P + M_L + M_E)\,\frac{1}{1+\kappa(\text{IL}_{10})} \quad \longleftarrow \textsf{synthesis}$$
$$- \mu_C\,\text{TNF}_\alpha \quad \longleftarrow \textsf{decay}$$

$$\dot{\text{IFN}}_\alpha = \rho_{\text{IFN}_\alpha}\,(M_L + M_E) \quad \longleftarrow \textsf{synthesis}$$
$$- \mu_C\,\text{IFN}_\alpha \quad \longleftarrow \textsf{decay}$$

The adaptive antiviral cytokine IFN_γ is synthesised by cells of the cellular response (R_c) and natural killers (NK). This synthesis is auto-amplified. It is also amplified by IFN_α and TNF_α, in synergy with IL_{12}, and inhibited by IL_{10} and TGF_β. IFN_γ is also an immuno-regulatory cytokine and it orients the adaptive response towards the cellular response [16–18, 20–23].

$$\dot{\text{IFN}}_\gamma = \rho_{\text{IFN}_\gamma}\,(R_c + NK)\,\frac{[1+\kappa(\text{IFN}_\gamma)]\,[1+\kappa(\text{IL}_{12},\text{TNF}_\alpha)]\,[1+\kappa(\text{IL}_{12},\text{IFN}_\alpha)]}{[1+\kappa(\text{IL}_{10})]\,[1+\kappa(\text{TGF}_\beta)]} \quad \longleftarrow \textsf{synthesis}$$
$$- \mu_C\,\text{IFN}_\gamma \quad \longleftarrow \textsf{decay}$$

Immuno-regulatory cytokines [12–18] The immuno-regulatory cytokines IL_{12}, IFN_γ (see above), IL_{10} and TGF_β have various functions, in particular the regulation of the adaptive immune response. They also regulate the recruitment of macrophages and natural killers, the phagocytosis and infection, as well as the cytokine syntheses. They are produced by cells of the innate and adaptive response.

IL_{12} co-amplifies the recruitment of macrophages, activates the natural killers and orients the adaptive response towards the cellular response. It is synthesised by activated macrophages. Its synthesis is inhibited by IL_{10}.

$$\dot{\text{IL}}_{12} = \rho_{\text{IL}_{12}}\,(M_P + M_L + M_E)\,\frac{1}{1+\kappa(\text{IL}_{10})} \quad \longleftarrow \textsf{synthesis}$$
$$- \mu_C\,\text{IL}_{12} \quad \longleftarrow \textsf{decay}$$

IL_{10} and TGF_β are both immuno-modulatory cytokines. IL_{10} inhibits the natural killer activation and the phagocytosis, it amplifies the macrophage permissiveness and it orients the adaptive response towards the humoral and regulatory responses. IL_{10} is synthesised by activated macrophages and cells of the regulatory (R_r) and humoral (R_h) responses. Its synthesis by macrophages and R_r is amplified by TGF_β, whereas its synthesis by R_h is auto-amplified and inhibited by TGF_β.

$$\dot{\text{IL}}_{10} = \rho_{\text{IL}_{10}}\left((M_P + M_L + M_E + R_r)\,[1+\kappa(\text{TGF}_\beta)] + R_h\,\frac{1+\kappa(\text{IL}_{10})}{1+\kappa(\text{TGF}_\beta)}\right) \quad \longleftarrow \textsf{synthesis}$$
$$- \mu_C\,\text{IL}_{10} \quad \longleftarrow \textsf{decay}$$

TGF_β inhibits the phagocytosis and macrophage permissiveness and orients the adaptive response towards the regulatory response. In the model, we neglected the synthesis of TGF_β by activated macrophages, so it is only synthesised by cells of the regulatory response.

$$\dot{\text{TGF}}_\beta = \rho_{\text{TGF}_\beta}\,R_r \quad \longleftarrow \textsf{synthesis}$$
$$- \mu_C\,\text{TGF}_\beta \quad \longleftarrow \textsf{decay}$$

References

1. Gammack D, Ganguli S, Marino S, Segovia-Juarez J, Kirschner D (2005) Understanding the immune response in tuberculosis using different mathematical models and biological scales. Multiscale Modeling & Simulation 3: 312-345.

2. Marino S, Myers A, Flynn JL, Kirschner DE (2010) TNF and IL-10 are major factors in modulation of the phagocytic cell environment in lung and lymph node in tuberculosis: A next-generation two-compartmental model. Journal of Theoretical Biology 265: 586-598.

3. Wigginton JE, Kirschner D (2001) A model to predict cell-mediated immune regulatory mechanisms during human infection with Mycobacterium tuberculosis. The Journal of Immunology 166: 1951-1967.

4. Rouse BT, Sehrawat S (2010) Immunity and immunopathology to viruses: what decides the outcome? Nature Review Immunology 10: 514-526.

5. Choi C, Chae C (2002) Expression of tumour necrosis factor alpha is associated with apoptosis in lungs of pigs experimentally infected with porcine reproductive and respiratory syndrome virus. Research in Veterinary Science 72: 45-49.

6. Van Reeth K, Van Gucht S, Pensaert M (2002) In vivo studies on cytokine involvement during acute viral respiratory disease of swine: troublesome but rewarding. Veterinary Immunology and Immunopathology 87: 161-168.

7. Roth AJ, Thacker EL (2009) Immune sytem. In: Straw BE, Zimmerman JJ, D'Allaire S, Taylor DL, editors, Diseases of swine, Blackwell, chapter 2. Ninth edition, pp. 15-36.

8. Tosi MF (2005) Innate immune responses to infection. Journal of Allergy and Clinical Immunology 116: 241-249.

9. Thanawongnuwech R (1999) Pulmonary intravascular macrophages (PIMs): What do we know about their role in PRRSV infection in pigs. Wetchasan Sattawaphaet (The Thai journal of veterinary medicine) 29.

10. DeFranco AL, Locksley RM, Robertson M, Cunin R (2009) Immunité : la réponse immunitaire dans les maladies infectieuses et inflammatoires. DeBoeck, Bruxelles, 2nd edition.

11. Vidal SM, Khakoo SI, Biron CA (2011) Natural killer cell responses during viral infections: flexibility and conditioning of innate immunity by experience. Current Opinion in Virology 1: 497-512.

12. Bosch AATM, Biesbroek G, Trzcinski K, Sanders EAM, Bogaert D (2013) Viral and bacterial interactions in the upper respiratory tract. PLoS Pathogens 9: e1003057.

13. Borghetti P (2005) Cell-mediated immunity and viral infection in pig. In: PRRS fatti vs speculazioni. Parma, Italy: Università degli Studi di Parma, Dipartimento di Salute Animale, pp. 27-46.

14. Braciale TJ, Sun J, Kim TS (2012) Regulating the adaptive immune response to respiratory virus infection. Nature Reviews Immunology 12: 295-305.

15. Coquerelle C, Moser M (2010) DC subsets in positive and negative regulation of immunity. Immunological Reviews 234: 317-334.

16. Kidd P (2003) Th1/Th2 balance: the hypothesis, its limitations, and implications for health and disease. Alternative Medicine Review 8: 223–246.

17. LeRoith T, Ahmed SA (2012) Regulatory T cells and viral disease. In: Khatami M, editor, Inflammation, Chronic Diseases and Cancer: Cell and Molecular Biology, Immunology and Clinical Bases, InTech, chapter 6. pp. 121-144. doi:10.5772/28502.

18. Knosp CA, Johnston JA (2012) Regulation of CD4+ T-cell polarization by suppressor of cytokine signalling proteins. Immunology 135: 101-111.

19. Yates A, Bergmann C, Van Hemmen JL, Stark J, Callard R (2000) Cytokine-modulated regulation of helper T cell populations. Journal of Theoretical Biology 206: 539-560.

20. Lopez-Fuertes L, Campos E, Domenech N, Ezquerra A, Castro JM, et al. (1999) Porcine reproductive and respiratory syndrome (PRRS) virus down-modulates TNF-α production in infected macrophages. Virus Research 69: 41-46.

21. Zimmerman J, Benfield DA, Murtaugh MP, Osorio F, Stevenson GW, et al. (2006) Porcine reproductive and respiratory syndrome virus (porcine arterivirus). In: Straw BE, Zimmerman JJ, D'Allaire S, Taylor DL, editors, Diseases of swine, Blackwell, chapter 24. Ninth edition, pp. 387-418.

22. Molina RM, Cha SH, Chittick W, Lawson S, Murtaugh MP, et al. (2008) Immune response against porcine reproductive and respiratory syndrome virus during acute and chronic infection. Veterinary immunology and immunopathology 126: 283-292.

23. Lunney JK, Fritz ER, Reecy JM, andr Elizabeth Prucnal DK, Molina R, et al. (2010) Interleukin-8, Interleukin-1β, and Interferon-γ levels are linked to PRRS virus clearance. Viral Immunology 23: 127-134.

24. Thanawongnuwech R, Rungsipipat A, Disatian S, Saiyasombat R, Napakanaporn S, et al. (2003) Immunohistochemical staining of IFN$_\gamma$ positive cells in porcine reproductive and respiratory syndrome virus-infected lungs. Veterinary Immunology and Immunopathology 91: 73-77.

25. Gómez-Laguna J, Salguero FJ, Pallarés FJ, Carrasco L (2013) Immunopathogenesis of porcine reproductive and respiratory syndrome in the respiratory tract of pigs. The Veterinary Journal 195: 148-155.

26. Albina E (2000) Le point sur le dernier-né des Arterivirus : le virus du syndrome dysgénésique et respiratoire procin (SDRP). Virologie 4: 113-121.

27. Murtaugh MP (2005) PRRSV/host interaction. In: PRRS fatti vs speculazioni. Parma, Italy: Università degli Studi di Parma, Dipartimento di Salute Animale, pp. 73-80.

28. Miller LC, Laegreid WW, Bono JL, Chitko-McKown CG, Fox JM (2004) Interferon type I response in porcine reproductive and respiratory syndrome virus-infected MARC-145 cells. Archives of Virology 149: 2453-2463.

29. Gimeno M, Darwich L, Díaz I, de la Torre E, Pujols J, et al. (2011) Cytokine profiles and phenotype regulation of antigen presenting cells by genotype-I porcine reproductive and respiratory syndrome virus isolates. Veterinary Research 42.

30. Chang HC, Peng YT, Chang HL, Chaung HC, Chung WB (2008) Phenotypic and functional modulation of bone marrow-derived dendritic cells by porcine reproductive and respiratory syndrome virus. Veterinary Microbiology 129: 281-293.

31. Peng YT, Chaung HC, Chang HL, Chang HC, Chung WB (2009) Modulations of phenotype and cytokine expression of porcine bone marrow-derived dendritic cells by porcine reproductive and respiratory syndrome virus. Veterinary Microbiology 136: 359-365.

32. Yoo D, Song C, Sun Y, Du Y, Kim O, et al. (2010) Modulation of host cell responses and evasion strategies for porcine reproductive and respiratory syndrome virus. Virus Research 154: 48-60.

33. Flores-Mendoza L, Silva-Campa E, Reséndiz M, Osorio FA, Hernndez J (2008) Porcine reproductive and respiratory syndrome virus infects mature porcine dendritic cells and up-regulates interleukin-10 production. Clinical and Vaccine Immunology 15: 720-725.

34. Suradhat S, Thanawongnuwech R, Poovorawan Y (2003) Upregulation of IL-10 gene expression in porcine peripheral blood mononuclear cells by porcine reproductive and respiratory syndrome. Journal of General Virology 84: 453-459.

35. Moore KW, de Waal Malefyt R, Coffman RL, O'Garra A (2001) Interleukin-10 and the interleukin-10 receptor. Annual Review of Immunology 19: 683-765.

36. Darwich L, Díaz I, Mateu E (2010) Certainties, doubts and hypotheses in porcine reproductive and respiratory syndrome virus immunobiology. Virus Research 154: 123-132.

37. Murtaugh MP, Xiao Z, Zuckermann F (2002) Immunological responses of swine to porcine reproductive and respiratory syndrome virus infection. Viral immunology 15: 533-547.

38. Gómez-Laguna J, Salguero FJ, Barranco I, Pallares FJ, Rodriguez-Gomez IM, et al. (2009) Cytokine expression by macrophages in the lung of pigs infected with the porcine reproductive and respiratory syndrome virus. Journal of Comparative Pathology 142: 51-60.

39. Underhill DM, Goodridge HS (2012) Information processing during phagocytosis. Nature Reviews Immunology 12: 492-502.

40. Akira S, Takeda K, Kaisho T (2001) Toll-like receptors: critical proteins linking innate and acquired immunity. Nature immunology 2: 675-680.

41. Sen GC (2001) Viruses and interferons. Annual review of Microbiology 55: 255-281.
